# Comparative plastome analysis and plastomic phylogeny of Moraceae with expanded species-level sampling

**DOI:** 10.1186/s12870-026-09508-4

**Published:** 2026-07-15

**Authors:** Hui-Long Li, Wen Deng, Chen-Xuan Yang, Wei Gu, Cheng Liu, Da-Cai Zhang, Jian Wang, Ting-Shuang Yi, Rong Zhang

**Affiliations:** 1 Southwest Forestry University, Kunming, 650224 China; 2 Germplasm Bank of Wild Species & Yunnan Key Laboratory of Crop Wild Relatives Omics, Kunming Institute of Botany, Chinese Academy of Sciences, Kunming, 650201 China; 3 College of Biology and Food Engineering, Southwest Forestry University, Kunming, 650224 China; 4 Department of the Environment, Tourism, Science and Innovation, Queensland Herbarium & Biodiversity Science, Brisbane Botanic Gardens, Mt Coot-Tha Road, Toowong, QLD 4066 Australia; 5 Queensland Alliance for Agriculture and Food Innovation, The University of Queensland, St Lucia, QLD 4072 Australia

**Keywords:** Comparative genomics, Genome structure, Molecular markers, Phylogenetic analysis, Plastome genomics

## Abstract

**Background:**

Moraceae is an economically and ecologically important angiosperm family. Although recent nuclear and plastid phylogenomic studies have greatly improved the systematic framework of Moraceae, structural evolution and lineage-specific variation of plastomes remain insufficiently evaluated under dense complete-plastome sampling. To address these issues, we conducted a plastome-based phylogenetic and comparative genomic analysis using 140 complete plastomes (49 newly sequenced and 91 publicly available), representing all seven tribes and 17 genera of Moraceae.

**Results:**

Moraceae plastomes were generally structurally conserved, although lineage-specific variation in inverted repeat (IR) boundaries was detected, particularly in *Artocarpus* and *Ficus*. Nucleotide diversity analyses identified several highly variable non-coding regions (including *ndhC-trnV(UAC)*, *ndhD-psaC*, *psbI-trnS(GCU)*, and *trnL(UAG)-ccsA*) and protein-coding genes (*matK*, *rps11*, *ndhF*, *rps15*, and *ycf1*), which may serve as candidate molecular markers for phylogenetic reconstruction and species identification in Moraceae. Analyses of repeat sequences and codon usage revealed a balance between structural conservation and sequence variability. Selection pressure analyses indicated that most plastid genes are under purifying selection, with a small number of genes showing signals consistent with positive selection in specific lineages, including *rbcL* in *Morus*, and *clpP* and *rps19* in *Ficus*. Phylogenetic reconstruction based on complete plastome sequences strongly supported the monophyly of all seven recognized tribes of Moraceae, recovered the non-monophyly of *Streblus*, and placed *Maclura* within Chlorophoreae, consistent with recent nuclear and plastid phylogenomic frameworks.

**Conclusion:**

Because plastomes represent a single, non-recombining organellar genome, our phylogenetic results should be interpreted as a plastome-based topology rather than a comprehensive species-tree reconstruction. This study provides an expanded species-level complete-plastome resource and phylogenetic framework for Moraceae, refines understanding of IR boundary evolution, and improves the robustness of candidate molecular markers for future systematic and species-identification studies.

**Supplementary Information:**

The online version contains supplementary material available at https://doi.org/10.1186/s12870-026-09508-4.

## Introduction

The plastid genome (plastome) is a highly conserved, circular molecule essential for photosynthesis, typically organized into a large single-copy (LSC) region, a small single-copy (SSC) region, and a pair of inverted repeats (IRs). Although generally conserved, lineage-specific structural and sequence compositional variations have accumulated over macroevolutionary timescales. Specifically, expansions and contractions of the IR regions are major contributors to plastome size variation and structural differentiation [1]. Moreover, repetitive sequences, including dispersed and tandem repeats, may facilitate recombination-mediated plastome rearrangements and contribute to lineage-specific structural variation [2, 3]. These structural changes provide phylogenetic signal and may be associated with functional divergence of photosynthesis-related genes, although direct links to adaptive evolution remain to be further tested [4, 5]. Consequently, comparative plastid genomics has become a fundamental tool for resolving deep phylogenetic relationships and exploring plant evolutionary mechanisms.

The family Moraceae (Rosales) comprises approximately 1,140 species in 47 genera [6–10]. It includes ecologically and economically important genera such as *Ficus*, *Morus*, *Artocarpus*, and *Maclura* [11–14]. A framework of seven tribes—Chlorophoreae, Artocarpeae, Moreae, Parartocarpeae, Dorstenieae, Ficeae, and Antiarideae—is now widely accepted [15–17]. Recent nuclear and plastid phylogenomic studies have further improved the systematic understanding of Moraceae. In particular, Yang et al. [18] employed extensive Hyb-Seq data from both nuclear and plastid loci and provided a comprehensive nuclear and plastid phylogenomic framework for the family, with broad generic sampling and explicit discussion of phylogenomic conflict, introgression, and incomplete lineage sorting. This framework provides an essential reference for interpreting plastome-based relationships. However, complete plastome data remain valuable because they enable detailed analyses of plastome architecture, IR boundary dynamics, mutational hotspots, repeat variation, codon usage, and plastid gene selection. Therefore, a complete-plastome-focused study can complement Hyb-Seq-based phylogenomic frameworks by providing a structural and evolutionary perspective on the organellar genome [18]. At the generic level, recent phylogenomic and biogeographic studies have also improved the understanding of major Moraceae lineages, including *Brosimum* and allied genera [19] and *Morus* [20].

Previous complete-plastome-based studies, particularly the study by Zhou et al. [17], have provided important insights into plastome structure, mutational hotspots, DNA barcoding potential, codon usage, selection pressure, and phylogenetic relationships in Moraceae. However, available analyses remain limited by relatively sparse species-level sampling, which may affect the assessment of within-lineage plastome variation, the stability of candidate molecular markers, and the detection of lineage-specific structural changes. Therefore, an expanded plastome dataset with denser species sampling is still needed to refine our understanding of plastome evolution and plastome-based relationships across Moraceae [17].

Recent advances in high-throughput sequencing technologies have facilitated plastome-based phylogenetic and comparative genomic studies for four genera of Moraceae, i.e. *Ficus* [11], *Sorocea* [21], *Allaeanthus* and *Malaisia* [22]. These plastome-based studies represent some of the most detailed analyses currently available for individual genera of Moraceae, providing useful references for understanding phylogenetic relationships within Moraceae. They have revealed overall plastome conservation while identifying mutational hotspots and genes showing signals consistent with positive selection. However, these studies are still constrained by limited taxonomic sampling or uneven tribal coverage [23–25].

This study aims to complement recent nuclear and plastid phylogenomic frameworks by providing a complete-plastome-based assessment of Moraceae evolution under expanded species-level sampling. Specifically, our objectives are to: (i) characterize plastome structural variation across Moraceae, with particular emphasis on IR boundary dynamics; (ii) identify highly variable plastome regions as candidate molecular markers for phylogenetic reconstruction and species identification; and (iii) reconstruct a plastome-based topology and compare it with recent phylogenomic frameworks, particularly with respect to tribal relationships and some generic placements, such as those of *Maclura* and *Streblus*.

## Materials and methods

### Sampling and sequencing

A total of 140 samples representing 17 genera and 134 species across all seven tribes of Moraceae were analyzed in this study. The dataset comprises 49 newly sequenced plastomes (Table S1), 91 previously published Moraceae plastome sequences (Table S2), and three outgroup plastomes downloaded from GenBank. The newly sequenced samples were obtained from fresh leaves, silica gel-dried leaves, and herbarium specimens. Their herbarium voucher information and accession numbers are provided in Table S1. For the new samples, total genomic DNA was extracted from fresh or silica gel-dried leaf material using a modified CTAB protocol. Following Illumina's technical guide, high-quality DNA was used to construct paired-end libraries with the VAHTS Universal Plus DNA Library Prep Kit for Illumina V2 (Vazyme, Nanjing, China; ND627-C1-P1) following the manufacturer’s protocols and sequenced on a DNBSEQ-T7 platform (Berry Genomics, Beijing, China).

### Plastome assembly, annotation, and comparative analysis

Plastomes were assembled using GetOrganelle v1.7.7 [26]. Annotation was performed using a multi-step approach: all 143 plastomes were first annotated using PGA v2.0 [27, 28], quality-assessed with the check_annotations.py script [26], and further validated using GeSeq [29] before final manual curation in Geneious v9.1.8 [30]. All 143 checked plastomes were used for phylogenetic analysis. The 127 plastomes were finally used for all subsequent analyses, excluding 10 plastomes containing gap nucleotides and plastomes of three duplicated species and three outgroups. Plastome maps were visualized using the online tool OGDRAW v1.3.1 [31]. Whole-genome synteny analyses were conducted with the Mauve plugin in Geneious using the progressiveMauve algorithm [32]. Comparative analyses were performed using mVISTA [33] under the shuffle-LAGAN alignment algorithm, with *Malaisia scandens* (PP728628) as the reference. A total of 25 highly divergent plastid genomes from 17 genera were selected for mVISTA analysis.

### Phylogenetic analysis

Protein-coding genes (PCGs) and intergenic spacer regions (IGSs) of 143 plastomes were automatically extracted from GenBank files using the script "get_annotated_regions_from_gb.py" implemented in PlastidHub [28] and aligned separately using MAFFT v7.409 [34] with default parameters, and poorly aligned regions were trimmed with trimAl v1.4 [35] under the "automated1" algorithm. The resulting alignments were concatenated into three datasets: PCGs-only, IGSs-only, and a concatenated whole-genome matrix (Full-Con), using the "concatenate" function in PhyloSuite v1.0 [36]. Maximum likelihood (ML) trees for each matrix were reconstructed using IQ-TREE v2.2.0 [37] implemented in PhyloSuite. The best-fit evolutionary model for each matrix was automatically selected by the "auto" function of PhyloSuite, which calls ModelFinder [38] to screen optimal models under Bayesian information criterion (BIC). Branch supports were evaluated with 1,000 bootstrap replicates. Three species, *Girardinia bullosa* (Urticaceae), *Parasponia rugosa* (Cannabaceae), and *Ulmus lamellosa* (Ulmaceae) were selected as outgroups.

### IR boundary analysis and nucleotide diversity

IR boundary shifts among the 127 plastomes were examined using the online tool CPJSdraw [39], visualizing structural variation at the junctions between LSC/IRb, IRb/SSC, SSC/IRa, and IRa/LSC regions. Nucleotide diversity (Pi) for PCGs and IGSs was calculated with the "Pi" module in CPStools v1.2 [40]. Prior to analysis, all degenerate nucleotides were converted to "N". The phylogeny reconstructed from the complete plastome matrix (the "Full-Con" tree) had the highest overall support and was therefore used as the reference tree. IR boundary patterns were classified according to gene arrangements at the four junctions: JLB (LSC/IRb), JSB (IRb/SSC), JSA (SSC/IRa), and JLA (IRa/LSC). Based on the genes involved at these junctions, the IR boundary patterns were assigned to four major types, which were further divided into finer subtypes according to the relative positions of junction-associated genes. Ancestral state reconstruction of IR boundary subtypes was conducted on the phylogenetic tree using a maximum likelihood method as implemented in the R package phytools [41, 42]. Model fitting was performed using the Mk model with an equal-rates (ER) assumption, and alternative symmetric (SYM) and all-rates-different (ARD) models were also evaluated. IR boundary subtypes were treated as discrete character states, with the 11 observed subtypes (I-1 to I-8, II-1, III-1, and IV-1) encoded as categorical factor levels and assigned to the corresponding tips of the phylogeny. Model selection was based on Akaike information criterion (AIC). Marginal ancestral state probabilities at internal nodes were estimated using the "ancr" function and visualized as pie charts on the phylogeny, while tip states were mapped according to the observed IR subtype of each taxon.

### Plastome codon usage bias and repeat sequence identification

Codon usage bias was analyzed for all PCGs across 127 Moraceae plastomes using CodonW v1.4.2 [43] to calculate codon counts and the relative synonymous codon usage (RSCU). An RSCU value > 1.0, < 1.0, or = 1.0 indicates codon usage frequencies that are higher than expected, lower than expected, or neutral, respectively. Additional codon usage statistics were generated using the CPStools v1.2 [40]. Simple sequence repeats (SSRs) were identified using the SSR module with the following thresholds: mono-nucleotide ≥ 10, di-nucleotide ≥ 5, tri-nucleotide ≥ 4, and tetra-, penta-, and hexa-nucleotide repeats ≥ 3. Long repeat sequences (forward, palindromic, reverse, and complementary) were identified using REPuter [44], with a minimum repeat length of 30 bp and a Hamming distance of 3.

### Selection pressure analyses

PCGs were aligned in codon-aware mode using the MAFFT plugin in PhyloSuite v2.0 [36], and the alignments were converted to PML format. All 79 plastid coding genes of 123 Moraceae taxa (four plastomes lacking *rpl22* were excluded) were aligned using MAFFT with default parameters. A maximum likelihood (ML) phylogenetic tree was reconstructed using IQ-TREE v2.2.0 [37] with 1,000 bootstrap replicates. Using this ML tree and the PML-formatted alignments, the nonsynonymous (dN) to synonymous (dS) substitution rate ratio (dN/dS) for each plastid gene was estimated with CodeML in PAML v4.10.7 [45]. Selection pressure was analyzed using branch-specific models: the one-ratio model (M0) assumes uniform selection pressure across all branches (parameters: model = 0, NSsites = 0), and the two-ratio model (M2) allows foreground branches to have different selection pressures from background branches. Three ecologically distinctive lineages were selected as foreground branches for M2 model analysis: *Ficus* (notable for its high species richness, syconium inflorescence, and obligate fig–wasp mutualism), *Morus* (characterized by broad economic importance and global environmental adaptability), and *Artocarpus* (tropical large-fruited trees with rapid growth and prominent fruit development); the remaining Moraceae species were treated as background branches. Models were configured (M0: model = 0, NSsites = 0; M2: model = 2, NSsites = 0) and compared via likelihood ratio test (LRT). Foreground branches were designated using EasyCodeML v1.0 [46], with all parameters set according to the PAML manual.

## Results

### Plastome features of Moraceae species

We assembled and annotated 140 Moraceae plastomes. All genomes exhibited the typical circular quadripartite structure, consisting of an LSC region, an SSC region, and a pair of IR regions (Fig. S1). The length of the 49 newly assembled plastomes ranged from 159,074 bp (*Morus alba*) to 162,493 bp (*Streblus tonkinensis*). The lengths of the IR, LSC, and SSC regions varied by 425 bp (from 25,585 bp in *Taxotrophis zeylanica* to 26,010 bp in *Fatoua villosa*), 2,656 bp (from 87,883 bp in *Morus alba* to 90,539 bp in *Streblus tonkinensis*), and 617 bp (from 19,693 bp in *Taxotrophis ilicifolia* to 20,310 bp in *Maclura fruticosa*). Overall GC content ranged from 35.31% (*Dorstenia elata*) to 36.2% (*Morus alba*). After excluding one IR copy, the number of annotated genes ranged from 110 to 111, comprising 78–79 protein-coding genes, 28 tRNA genes, and 4 rRNA genes (Table S3). Gene loss was rare (8/140 genomes), occurring only in the following taxa: *rpl22* was absent in *Broussonetia* × *hanjiana*, *Broussonetia kaempferi*, *Broussonetia monoica*, and *Broussonetia* × *kazinoki*, and *trnH-GUG* was lost in *Ficus lyrata*, *Ficus benjamina*, *Ficus virens*, and *Ficus racemosa*. Additionally, *rpl2* was annotated as a pseudogene in the IRa region of *Ficus simplicissima* and *Taxotrophis ilicifolia*.

### IR boundary dynamics

**Fig. 1:**
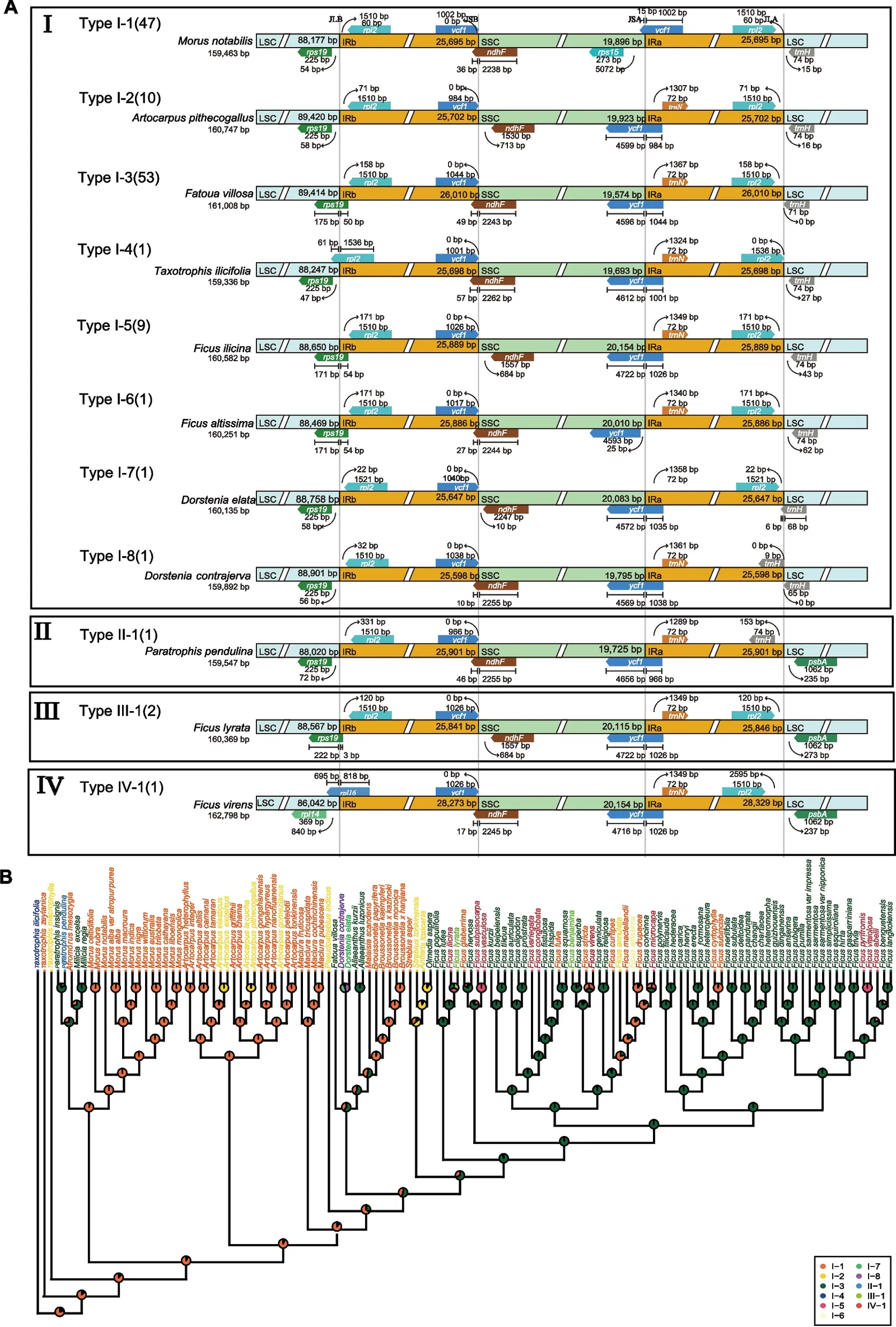
IR boundary types and ancestral state reconstruction in Moraceae plastomes. **A** Schematic diagram of four boundary types (with 11 subtypes) between LSC, SSC, and IR regions among 127 Moraceae plastomes. Abbreviations: IR, inverted repeat; LSC, large single-copy; SSC, small single-copy. **B** Maximum-likelihood ancestral state reconstruction of IR boundary subtypes in Moraceae. IR subtypes were coded as 11 discrete states (I-1 to I-8, II-1, III-1, and IV-1). Colored tip labels indicate the observed IR subtype of each species. Pie charts at internal nodes represent the marginal probabilities of ancestral states. Branch lengths are proportional to genetic distance

Four major IR boundary types (I-IV) were identified (Fig. 1A). Type I was overwhelmingly dominant (123/127 plastomes) and was further divided into eight subtypes (I-1 to I-8), whereas Types II-IV were rare (4/127). Within Type I, subtypes I-3 (53 plastomes across six genera) and I-1 (47 plastomes across nine genera) were the most widespread, while the remaining subtypes were restricted to a few or single species. These subtypes were primarily distinguished by the positions of IR boundaries at the four junctions (JLB, JSB, JSA, and JLA). In most plastomes, JLB was located within or adjacent to *rps19*, JSB was typically positioned within or near *ndhF*, and JSA commonly spanned *ycf1*, whereas JLA showed comparatively higher variability but was generally located between *rpl2* and *trnH-GUG*. The three minor types (II-IV) exhibited strong lineage specificity. Type II was restricted to *Paratrophis pendulina*, Type III occurred only in *Ficus benjamina* and *Ficus lyrata*, and Type IV was unique to *Ficus virens*. These types were characterized by distinct boundary shifts, including displacement of JLA toward *psbA* and, in Type IV, a pronounced IR expansion placing JLB within *rpl16*.

Examination of IR junctions revealed consistent patterns of IR expansion and contraction (Fig. S2). At the LSC/IRb boundary (JLB), IRb expansion into the LSC was common, particularly in *Ficus*, where the 3' end of *rps19* extended 3–208 bp into IRb (most commonly ~ 54 bp). In contrast, IR contraction was observed in *Taxotrophis ilicifolia* (subtype I-4), where part of *rpl2* extended into the LSC. At the IRb/SSC boundary (JSB), variation was mainly associated with IR contraction, resulting in *ndhF* being entirely located within the SSC in several species (e.g., *Ficus abelii* and *Antiaris toxicaria*). At the SSC/IRa boundary (JSA), *ycf1* typically spanned the junction. However, exceptions were observed in *Ficus altissima* (subtype I-6), where *ycf1* was entirely located within the SSC, and in *Morus notabilis*, where it was almost completely shifted into IRa. The IRa/LSC boundary (JLA) exhibited the highest variability, particularly involving *trnH-GUG*, which showed three main patterns: spanning the boundary (e.g., *Dorstenia*), entirely located within IRa (e.g., *Paratrophis pendulina*), or absent in several *Ficus* species.

Ancestral reconstruction of IR subtypes across 127 Moraceae plastomes revealed substantial variation with a clear phylogenetic signal (Fig. 1B). Several clades were predominantly characterized by subtypes I-1 and I-3, whereas IR subtypes at deep nodes were reconstructed with nearly equal marginal probabilities.

### Identification of divergent hotspot regions

**Fig. 2:**
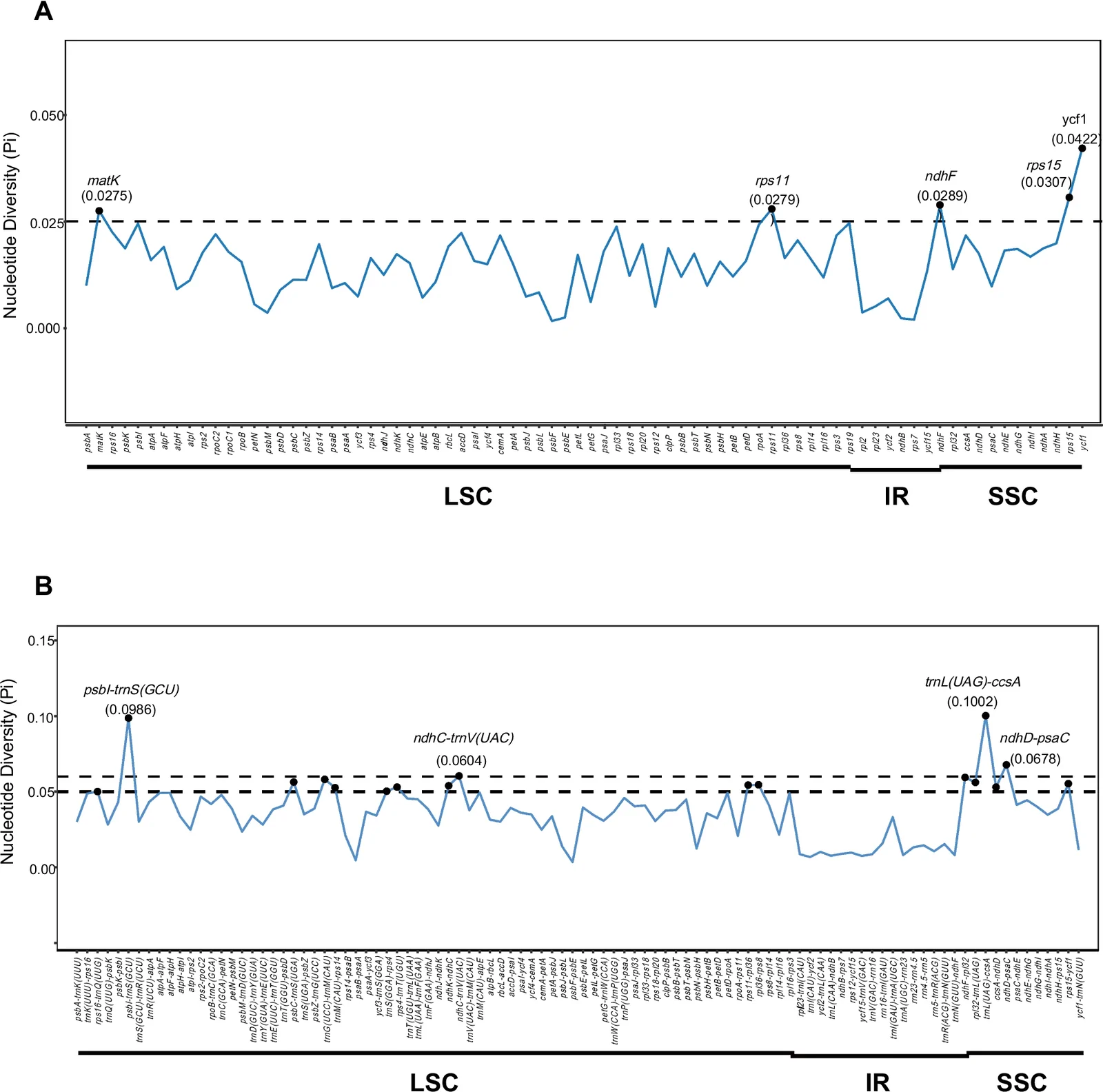
Nucleotide diversity (Pi) values of different regions of 127 Moraceae plastomes. **A** Pi values of protein-coding regions; **B** Pi values of non-coding regions

Nucleotide diversity was assessed across 127 Moraceae plastomes. Overall, diversity was higher in the single-copy regions than in the IR regions, and in non-coding regions than in coding regions (Fig. 2). Most protein-coding genes were highly conserved (Pi < 0.02), whereas *matK*, *rps11*, *ndhF*, *rps15*, and *ycf1* were more variable (Pi > 0.025), while *psbF*, *psbE*, *ndhB*, and *rps7* were nearly invariant (Pi ≈ 0). Non-coding regions showed higher diversity overall (Pi mostly 0.02–0.06), with *rps16-trnQ(UUG)*, *trnS(GGA)-rps4*, *trnM(CAU)-rps14*, *ccsA-ndhD*, *rps4-trnT(UGU)*, *ndhK-ndhC*, *rps11-rpl36*, *rpl36-rps8*, *rps15-ycf1*, *rpl32-trnL(UAG)*, *psbC-trnS(UGA)*, *trnG(UCC)-trnM(CAU)*, *ndhF-rpl32*, *ndhC-trnV(UAC)*, *ndhD-psaC*, *psbI-trnS(GCU)*, and *trnL(UAG)-ccsA* exhibiting Pi > 0.05. Synteny analysis revealed that gene order and orientation were highly conserved across all genomes, with no large-scale inversions or translocations (Fig. S3). Whole-genome alignments visualized via mVISTA further confirmed that coding regions were highly conserved, while non-coding regions contained multiple variable hotspots (Fig. S4). Because these hotspots were identified from 127 plastomes, they provide a broader species-level assessment of candidate markers than those inferred from smaller datasets.

### Repeat analyses

**Fig. 3:**
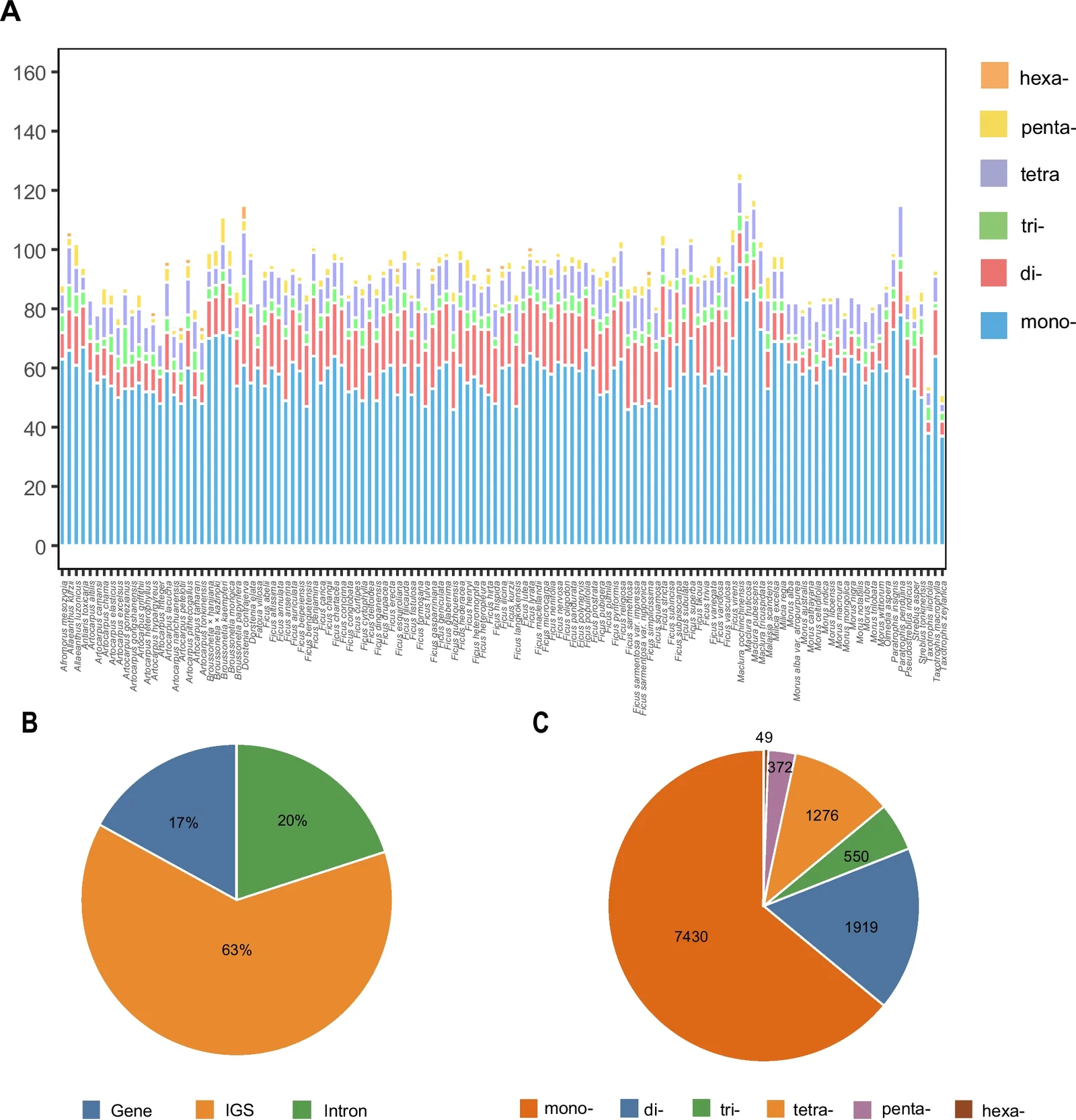
Distribution of simple sequence repeats (SSRs) in 127 plastomes of Moraceae. **A** Total numbers of SSRs detected in each species; **B** Distribution of SSRs in introns, coding sequences, and intergenic spacers (IGS); **C** Frequency of different SSR types

A total of 11,596 SSRs were identified across the 127 Moraceae plastomes. The number of SSRs per plastome varied substantially, ranging from 51 in *Taxotrophis zeylanica* to 126 SSRs in *Maclura cochinchinensis* (Fig. 3A). Overall, 63% of SSRs were located in non-coding regions (Fig. 3B). Mononucleotide repeats were the most abundant (64%), followed by dinucleotide (17%) and tetranucleotide repeats (11%), while trinucleotide repeats were relatively rare (5%), and pentanucleotide and hexanucleotide repeats were only detected in a subset of plastomes (Fig. 3C). Notably, several loci were SSR-rich, including *ycf1* (882 SSRs), *ndhF* (414), *rpl32-trnL(UAG)* (428), and *ndhF-rpl32* (301).

**Fig. 4:**
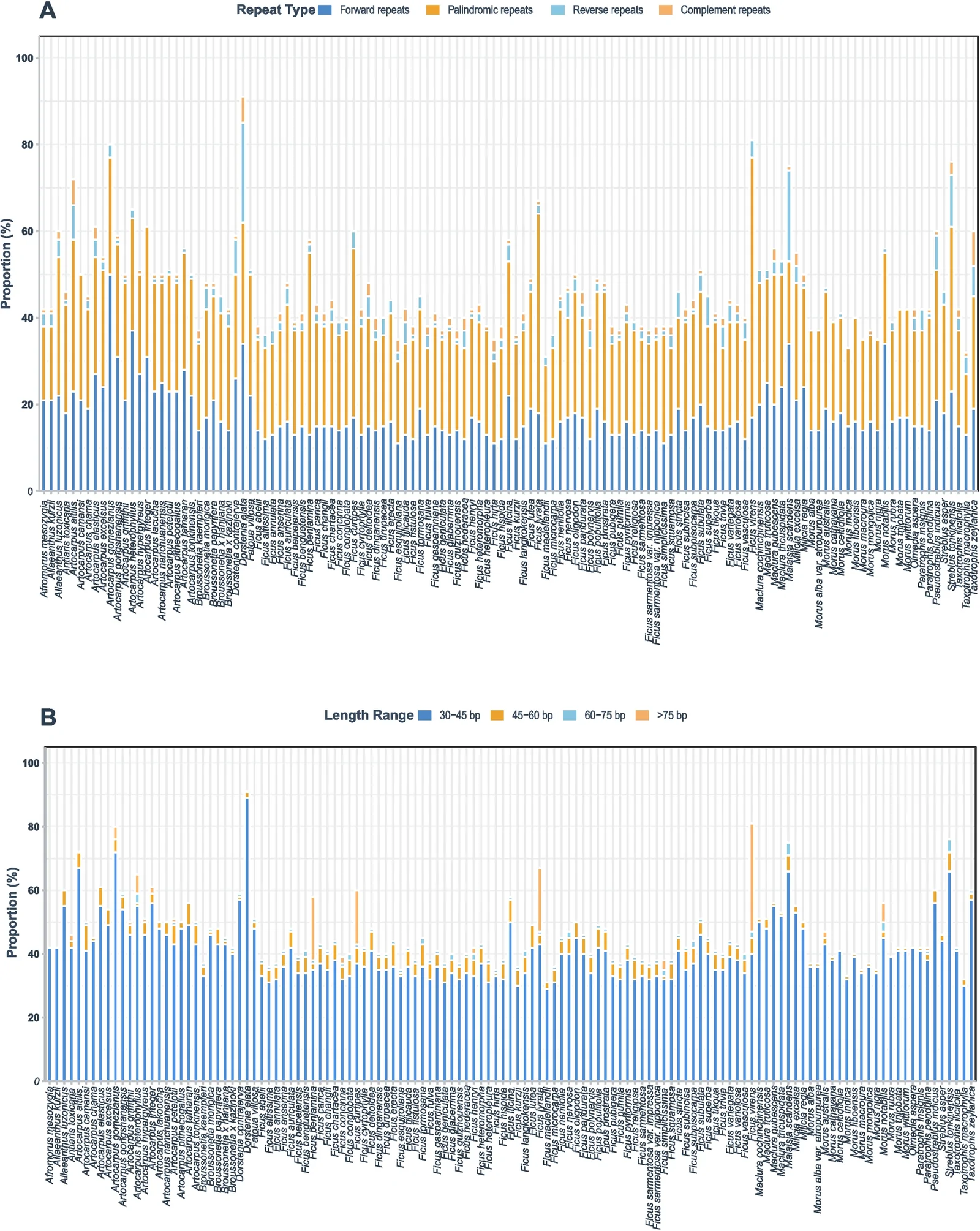
Analysis of long repeat sequences in 127 plastomes of Moraceae. **A** Types and their proportions of long repeat sequences identified in each species; **B** Length ranges and their proportions of long repeat sequences identified in each species

Analysis of long repeats identified 5,873 sequences, including 3,111 palindromic, 2,259 forward, 345 reverse, and 158 complementary repeats (Table S5). The lengths of these repeats ranged from 30–9,568 bp (palindromic), 30–144 bp (forward), 30–38 bp (complementary), and 30–71 bp (reverse). The number of long repeats per plastome ranged from 31 in *Ficus maclellandii* to 91 in *Dorstenia elata* (Fig. 4A). In repeat types, palindromic repeats were dominant (53%), followed by forward (38%), reverse (6%), and complementary repeats (3%) (Fig. 4B). In length ranges, the majority (88%) were 30–45 bp, while 8% were 45–60 bp and 4% were longer than 60 bp.

### Codon usage analyses

**Fig. 5:**
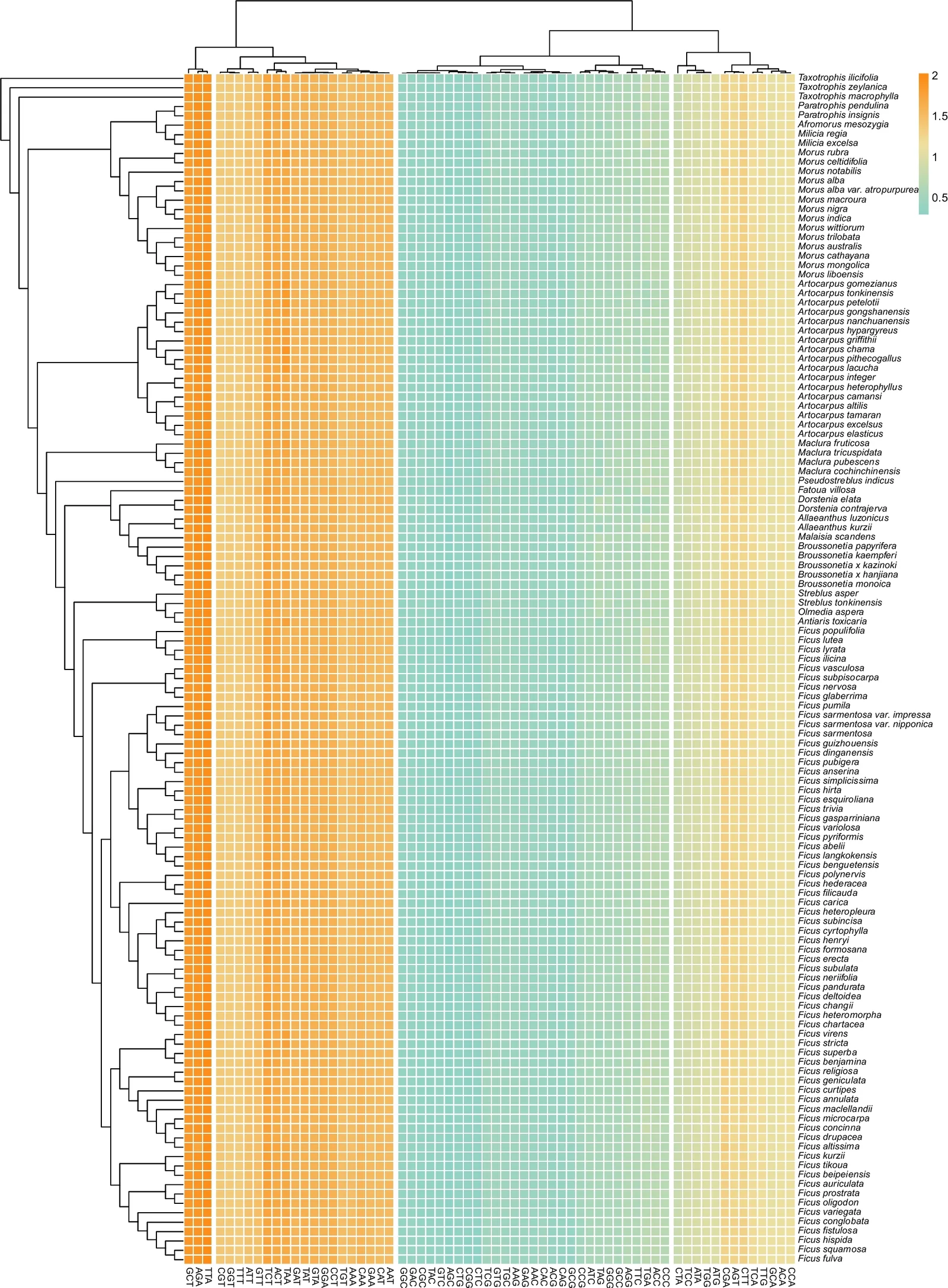
Relative synonymous codon usage (RSCU) analysis for protein-coding genes across 127 plastomes of Moraceae

Analysis of 127 Moraceae plastomes revealed that protein-coding regions contained from 15,879 codons in *Ficus altissima* to 18,277 in *Fatoua villosa*. The most abundant codon was GAA (Glu), occurring 655 to 789 times. Relative synonymous codon usage (RSCU) analysis revealed that TTA (Leu) exhibited the strongest preference, with an average RSCU value of ~ 1.96. Of the 30 codons with RSCU > 1.0, 29 ended with A or T. By contrast, ATG (Met) and TGG (Trp) showed no usage bias with RSCU values of 1.0 in all plastomes (Fig. 5).

### Phylogenetic inferences

**Fig. 6:**
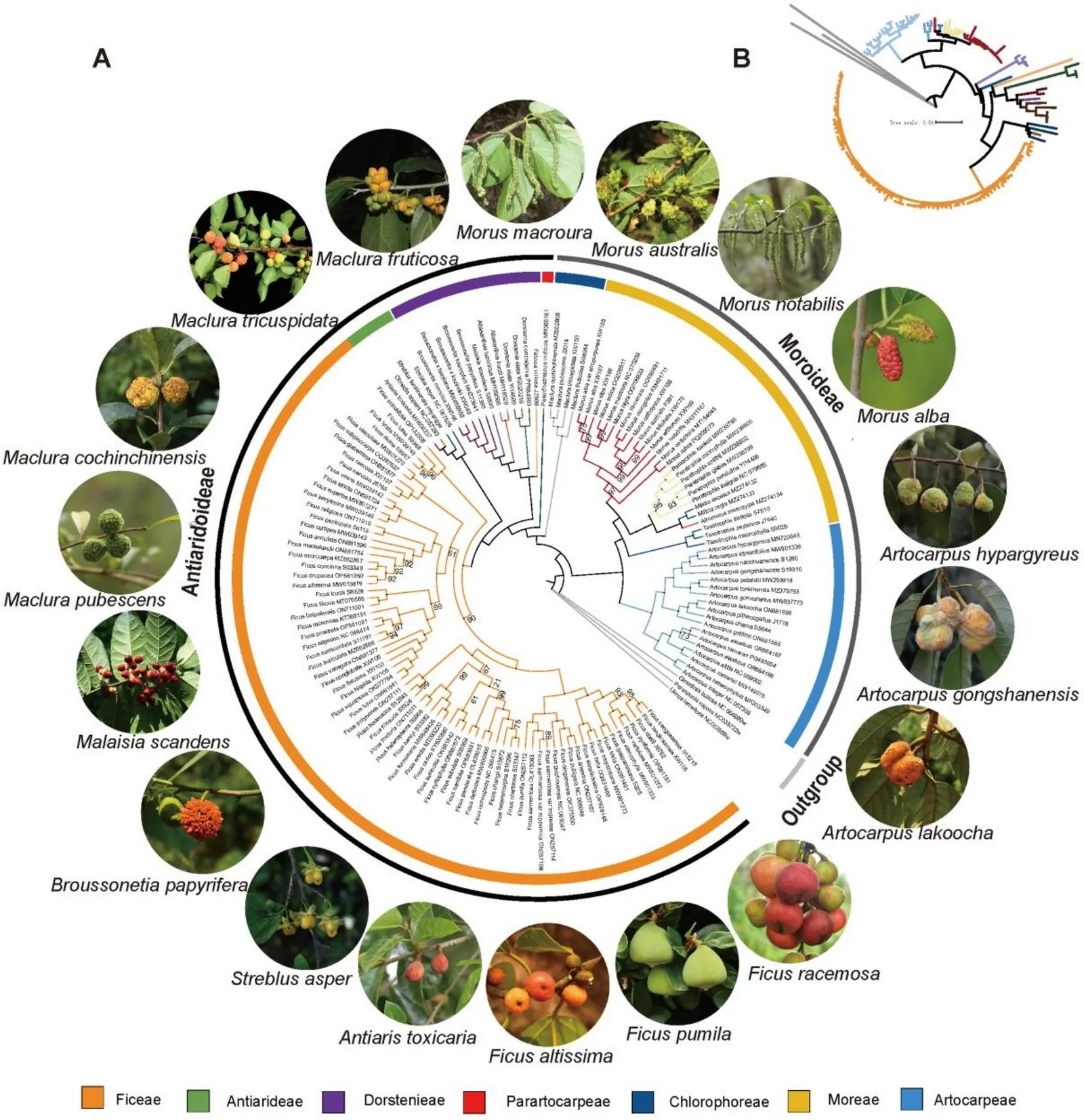
Maximum likelihood tree based on 143 plastome sequences, including 140 Moraceae plastomes and three outgroups. **A** A cladogram. Different branch colors represent different genera. The inner ring outside the phylogeny uses seven colors to present seven tribes, the outer ring uses varying gray shades to denote different subfamilies and the outgroups. **B** A phylogram. Different branch colors represent different genera. Bootstrap support values are shown only for nodes with < 100% support; all unlabeled nodes have 100% bootstrap support

Phylogenetic trees inferred from protein-coding genes (PCGs), intergenic spacers (IGS), and the complete plastome matrices were largely congruent with strong nodal support (mean bootstrap support: 95.8% for the complete plastome tree, 82.4% for the CDS tree, and 95.6% for the IGS tree). All three phylogenetic reconstructions recovered identical backbone topologies. The phylogenetic tree reconstructed from the complete plastome matrix had the highest overall support and was therefore illustrated (Fig. 6). The tree resolved Moraceae into two well-supported clades corresponding to subfamilies Moroideae and Antiaridoideae, and recovered all seven currently recognized tribes as monophyletic. Moroideae comprised Chlorophoreae (*Maclura*), Artocarpeae (*Artocarpus*), and Moreae (*Morus*, *Paratrophis*, *Milicia*, *Afromorus*, and *Taxotrophis*). Antiaridoideae included Parartocarpeae (*Pseudostreblus*), Dorstenieae (*Broussonetia*, *Malaisia*, *Dorstenia*, *Allaeanthus*, *Fatoua*), Ficeae (*Ficus*), and Antiarideae (*Antiaris*, *Streblus*, *Olmedia*). All sampled genera formed well-supported monophyletic clades except *Streblus*, which was consistently non-monophyletic across all analyses (Fig. S5A–B). Within *Ficus*, species assigned to the six traditional subgenera (*Sycomorus*, *Urostigma*, *Pharmacosycea*, *Ficus*, *Synoecia*, and *Sycidium*) were not recovered as monophyletic, with representatives of each subgenus split across multiple lineages (Fig. 6).

### Selection pressure analysis

**Fig. 7:**
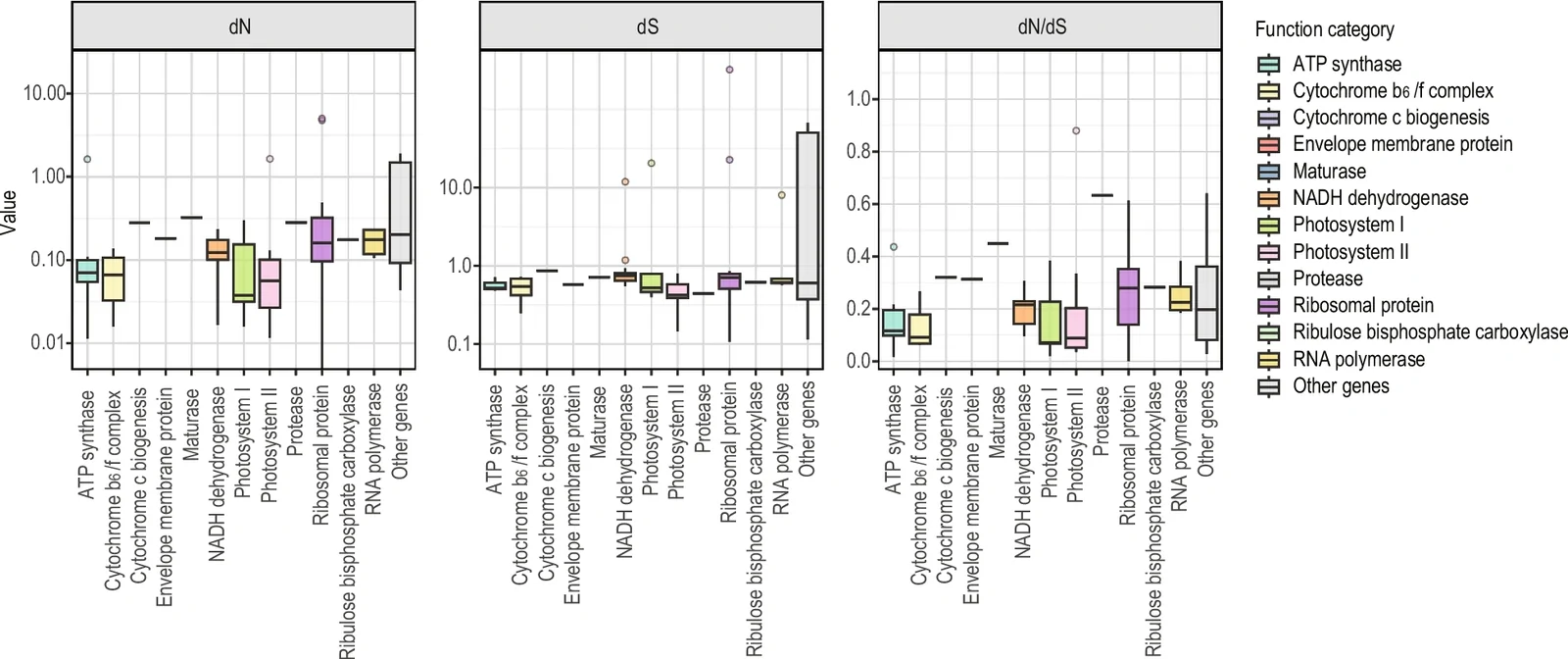
Nonsynonymous/synonymous substitution rate ratios (dN/dS) for protein-coding genes in 123 Moraceae plastomes. Genes were classified into functional categories based on their annotated biological functions in plastids, following standard plastome gene classification schemes (e.g., photosystem I and II, ATP synthase, electron transport components, ribosomal proteins, and transcription-related genes). Each box represents the interquartile range (IQR), with the horizontal line indicating the median value

Selection pressure analyses of 123 Moraceae plastomes using the M0 (one-ratio) model showed that the dN/dS (ω) values of 79 protein-coding genes ranged from 0.02 to 0.88, and functional categories presented variation but all ω values were < 1 (Fig. 7). These results indicate that plastid genes in Moraceae are predominantly under purifying selection at the family-wide scale, with no evidence of pervasive positive selection. To evaluate differences in selection pressure between foreground and background branches, the M2 (two-ratio) model was applied. Likelihood ratio tests (LRTs) indicated that, for most genes, the M0 model fit the data better than the M2 model when *Ficus*, *Morus*, and *Artocarpus* were designated as foreground branches (see Tables S7–S9). After excluding genes with extreme ω estimates (ω = 999 or ω = 0.0001), the M2 model fit the data better for a limited number of genes in specific foreground lineages (*P* < 0.05), with ω values > 1. Signals of positive selection were detected in *clpP* and *rps19* in *Ficus*, *rbcL* in *Morus*, and *clpP*, *rpl20*, *rps18*, *rps8*, and *ycf2* in *Artocarpus*. Overall, positive selection was restricted to a small subset of genes and lineages and was not pervasive across Moraceae.

## Discussion

### Plastome structural conservation and lineage-specific variation

Our analysis of 127 plastomes confirms that Moraceae plastomes maintain the highly conserved quadripartite structure typical of angiosperms, with genome size, GC content, and numbers of encoded genes falling within the typical range observed in land plants [47, 48]. These parameters are comparable to those of other terrestrial plant groups such as Rosaceae [49] and Malvaceae [50], further supporting the conservation of core plastome architecture and gene composition during seed plant evolution. This conservation may be associated with strong functional constraints on fundamental biological processes such as photosynthesis and energy metabolism [51].

We identified shifts at IR boundaries as the primary source of structural variation, revealing clear phylogenetic signals. These variations were classified into four major types and 11 subtypes, with patterns showing genus-level specificity (e.g., subtypes I-5, I-6, III, and IV in *Ficus*, subtypes I-7 and I-8 in *Dorstenia*). Notably, the IR expansion at the JLB was common in *Ficus*, while a unique contraction at the JLA involving *trnH-GUG* loss characterized some *Ficus* species. These lineage-specific IR dynamics, which may be associated with recombination between repeat sequences, represent a controlled mode of structural evolution without large-scale gene rearrangements, as evidenced by conserved synteny across the family. Similar lineage-associated IR boundary patterns have been reported in multiple angiosperm groups [52, 53].

Gene loss events in Moraceae plastomes appear to be relatively rare but show genus-specific patterns. Specifically, losses of *rpl22* and *trnH-GUG* were restricted to *Broussonetia* and *Ficus*, respectively, while pseudogenization of *rpl2* occurred only in a few species. These patterns align with broader angiosperm trends; for instance, the loss of *rpl22* has been independently reported in several families such as Passifloraceae [54], Rosaceae [55], Araceae [56], and Fabaceae [57, 58] and is often attributed to functional replacement or transfer to the nuclear genome. The plastid tRNA gene *trnH-GUG* is generally highly conserved across angiosperms, and its loss in a subset of *Ficus* species appears to be relatively uncommon, having been reported only in a few specialized lineages, such as *Saxifraga nigroglandulifera* [59] and *Olea europaea* subsp. *guanchica* [60], suggesting potential lineage-specific evolutionary trajectories for this gene. A notable finding is the absence of *infA* in all examined Moraceae plastomes, confirming and extending earlier observations from smaller samplings [17]. This indicates a systematic loss of *infA* within the family. The recurrent loss of *infA* has been widely documented across angiosperms [61, 62] and is commonly regarded as a representative example of plastid-to-nuclear gene transfer.

In conclusion, Moraceae plastomes are characterized by strong overall structural conservation with lineage-specific and constrained structural evolution. The evolutionary signature is primarily reflected in fine-tuning of the IR boundaries and the lineage-specific loss of a small number of genes.

### Identification of hypervariable regions as candidate molecular markers

The spatial distribution of sequence variation within plastomes provides an important basis for identifying molecular markers suitable for species delimitation and phylogenetic analyses [63, 64]. Overall, Moraceae plastomes conform to the general angiosperm pattern, with higher variability in single-copy and non-coding regions than in IR regions and coding regions. Therefore, marker development for this family should focus on identifying highly variable regions within otherwise conserved plastomes. Analysis of nucleotide diversity (Pi) identified several intergenic regions with elevated variation, making them promising candidates for Moraceae-specific DNA markers. This aligns with findings in other plant groups, such as *ndhC-trnV(UAC)* and other non-coding regions in *Schima* [65] and *ndhD-psaC* and additional intergenic regions in *Lindera aggregata* [66], supporting the broader utility of these highly variable non-coding regions for molecular marker development.

Among coding regions, *ycf1* represents one of the most informative loci, a status consistent with its validated utility across angiosperms in studies such as those on *Cicer* [67], *Delphinium* [68], and Moraceae [17]. Critically, our study further reveals structural variation in *ycf1* near the SSC/IRa boundary, indicating that it provides not only sequence-based variation but also complementary structural information, thereby enhancing its utility for phylogenetic inference and species identification. Additional candidate markers identified, such as *ndhF*, align with loci successfully applied in previous Moraceae plastome studies [19], reinforcing their validity and reliability as molecular markers.

Beyond sequence polymorphism, SSRs provide a complementary marker system due to their codominant inheritance and high polymorphism, which supports their wide application in plant population genetics and species identification [69–71]. For instance, SSR markers have been successfully developed and applied in *Lotus* [72]. In Moraceae plastomes, the distribution of SSRs shows a high degree of concordance with regions of elevated sequence variability, making these SSR-enriched regions a valuable complement to markers identified through nucleotide diversity. Critically, the convergence of evidence from independent analytical approaches strengthens the reliability of these candidate regions and enhances their potential utility in subsequent applications. By contrast, long repeat sequences and codon usage patterns offer more limited direct utility for marker screening, as they primarily reflect conserved structural and compositional features of the plastome [17, 73]. In Moraceae, the pattern of AT-driven codon usage bias is consistent with those observed across angiosperms [74, 75], indicating an absence of pronounced lineage-specific deviations in these compositional properties. Compared with candidate markers inferred from smaller datasets, hotspots identified from broader species-level sampling are less likely to reflect lineage-specific or taxon-sampling artifacts and may therefore provide more robust candidates for future Moraceae barcoding and species-identification studies.

### Selection pressure and potential adaptive evolution

Selection pressure analyses indicate that plastid genes of Moraceae are overall subject to strong purifying selection; however, a small number of genes exhibit lineage-specific signatures of positive selection in key lineages characterized by distinct ecological features or growth strategies. Similar patterns have been reported in previous studies of Moraceae. For example, Zhang et al. (2022) identified elevated *ω* (dN/dS) values for *rbcL* and *clpP* in *Ficus*, suggesting adaptive changes in these genes involved in various plastid biological processes [19]. Similarly, a broader plastome-wide analysis across all seven tribes of Moraceae found that while most genes are under purifying selection, a subset of genes shows lineage-dependent selection patterns [17]. Compared with broad family-level scans, the foreground-branch strategy used here allows selection-pressure variation to be interpreted in a more lineage-specific context, particularly for *Ficus*, *Morus*, and *Artocarpus*, which differ markedly in species richness, ecological associations, and growth strategies.

Positively selected genes identified in our study could be involved in fundamental plastid biological processes, including photosynthesis (*rbcL*), protein quality control (*clpP*), protein import (*ycf2*), and translation (*rps* and *rpl* gene families). The gene *rbcL* encodes the large subunit of Rubisco, the core enzyme of photosynthetic carbon fixation [76], whereas *clpP* participates in plastid protein quality control and stress responses [77]. *Ycf2* encodes a component of the protein import motor [78], and members of the *rps* and *rpl* gene families encode plastid ribosomal proteins involved in plastid translation [79]. Notably, previous studies have reported associations between plastome evolution, plastid gene selection, and adaptive evolution in other plant lineages [80, 81], although such interpretations remain debated and require experimental validation. Thus, these genes represent candidates for investigating potential lineage-specific functional adaptations, pending further experimental verification.

In the *Ficus* foreground branch, elevated ω values for *clpP* and *rps19* are consistent with previous studies [19], suggesting the possibility that plastid protein quality control-related processes may have experienced lineage-specific selective pressures. In the *Morus* foreground branch, elevated ω values for *rbcL* may reflect differential selective pressures on photosynthetic carbon fixation, though this requires functional validation. In the *Artocarpus* foreground branch, elevated ω values detected in *clpP*, *ycf2*, and several plastid ribosomal protein genes (*rpl20*, *rps18*, and *rps8*) largely overlap with patterns identified in comparative plastome analyses of *Artocarpus* and its close relatives [17], suggesting that plastid protein homeostasis, protein import, and translational machinery may represent targets of lineage-specific selection in large tropical woody plants, subject to future experimental confirmation. Overall, these lineage-specific patterns of elevated ω values may reflect differential selective pressures on key plastid functions across different Moraceae lineages.

It should be noted that statistical significance detected by PAML only reflects potential differences in selective pressure and cannot be directly interpreted as functional evidence of ecological adaptation [82, 83]. The association between plastid gene variation and species' adaptation to macro-ecological niches remains to be further validated through experimental approaches, including gene functional assays, population genetic analyses, and expression profiling under different environmental conditions [84]. Therefore, the discussion regarding the potential association of these genes with specific ecological contexts in this study is presented merely as a preliminary hypothesis, aiming to provide candidate genes and research directions for future investigations, rather than establishing definitive conclusions about adaptive evolution.

### Phylogenetic relationships in Moraceae

The plastome-based topology recovered here should be considered in light of recent nuclear and plastid phylogenomic studies of Moraceae. Yang et al. [18] provided a comprehensive family-wide framework based on extensive Hyb-Seq data from both nuclear and plastid loci and demonstrated that phylogenomic conflict is widespread in Moraceae, with introgression and incomplete lineage sorting contributing to discordance among nuclear genes and between nuclear and plastid genomes [18]. Compared with that study, our sampling is narrower at the generic level, and our phylogenetic inference is based exclusively on complete plastome sequences. Therefore, the topology recovered here should be interpreted as a plastome-based phylogeny rather than as a comprehensive species-tree estimate for Moraceae.

Despite this limitation, our complete-plastome analyses recovered a topology broadly consistent with recent systematic frameworks in several major respects [15, 17, 18]. All seven currently recognized tribes were resolved as monophyletic, consistent with recent nuclear phylogenomic inferences [18] and previously reported plastome-based phylogenies [17]. The placement of *Maclura* within Chlorophoreae and the close relationship between *Streblus* and Antiarideae were also consistent with recent phylogenomic and taxonomic studies [15, 18]. These results indicate that complete plastome data retain strong phylogenetic signal for plastid-level inference and provide a useful organellar perspective that complements multilocus nuclear phylogenomic frameworks.

All sampled *Maclura* species formed a well-supported clade corresponding to Chlorophoreae, whereas *Morus*, *Paratrophis*, *Milicia*, *Afromorus*, and *Taxotrophis* constituted the core lineage of Moreae. *Taxotrophis* was recovered as a strongly supported monophyletic lineage embedded within the core of Moreae, supporting its placement in this tribe. Although *Maclura* shares certain morphological similarities with several traditional genera of Moreae, it is consistently resolved outside the tribe, in agreement with recent taxonomic revisions that exclude it [15, 18].

Within Ficeae, *Ficus* has traditionally been divided into six subgenera (*Sycomorus*, *Urostigma*, *Pharmacosycea*, *Ficus*, *Synoecia*, and *Sycidium*), but these are not consistently recovered as monophyletic in the plastome-based phylogeny. This result is consistent with previous studies showing that relationships within *Ficus* vary with dataset type, taxon sampling, and analytical approach. Widespread discordance between plastid and nuclear phylogenies has been documented [16, 85] and is commonly attributed to processes such as hybridization, introgression, and incomplete lineage sorting [86]. Accordingly, the non-monophyly of subgenera inferred from plastome data should be interpreted cautiously and does not necessarily conflict with classifications based on nuclear or combined evidence.

The placements of *Pseudostreblus* and *Streblus* are likewise consistent with recent nuclear phylogenomic results. *Pseudostreblus* is recovered within Parartocarpeae and outside Moreae, in line with previous studies [17]. In contrast, *Streblus* is not monophyletic and is clearly separated from core Moreae, showing closer affinity to Antiarideae (formerly Olmedieae). This pattern agrees with nuclear phylogenomic evidence that excludes *Streblus* from Moreae and supports its transfer to Antiarideae [18], reinforcing the view that it does not belong to the core mulberry lineage.

At the generic level, expanded plastome sampling confirms the monophyly of *Morus* and supports its close relationships with genera such as *Paratrophis* and *Milicia*, consistent with recent studies [18, 25]. However, as only a single representative of *Pseudostreblus* is included here, broader sampling within Parartocarpeae, particularly of *Hullettia* and *Parartocarpus*, will be necessary to better resolve relationships within this tribe.

In summary, the broad consistency between our plastome-based topology and recent phylogenomic frameworks supports the utility of complete plastomes for plastid-level inference, but it should not be interpreted as evidence that plastome data alone can resolve the full species tree of Moraceae.

### Relationship to previous Moraceae plastome and phylogenomic studies

Recent studies have substantially advanced our understanding of Moraceae evolution from different genomic perspectives. Zhou et al. [17] provided an important complete-plastome-based analysis of Moraceae, including plastome structure, mutational hotspots, codon usage, repeat sequences, selection pressure, DNA barcoding potential, and plastome-based phylogeny. Yang et al. [18], in contrast, provided a broader nuclear and plastid Hyb-Seq phylogenomic framework and explicitly addressed phylogenomic conflict, introgression, and incomplete lineage sorting across the family. The present study does not replace either of these contributions. Instead, it extends previous complete-plastome studies by incorporating denser species-level sampling and by focusing on detailed plastome structural evolution.

The main contribution of our study lies in the generation and integration of 49 newly sequenced plastomes and the analysis of 140 complete Moraceae plastomes. This expanded dataset provides a stronger basis for assessing within-family plastome variation and reduces the possibility that candidate molecular markers are biased by limited taxon sampling. In addition, we provide a detailed analysis of IR boundary evolution by classifying Moraceae plastomes into four major IR boundary types and 11 subtypes and by reconstructing ancestral IR boundary states across the family. This analysis reveals lineage-specific IR boundary dynamics that are not fully captured by general comparisons of plastome structure alone.

Therefore, although several analytical categories overlap with previous plastome studies, the present study refines and strengthens the complete-plastome-based understanding of Moraceae. Its value lies not in replacing the broader phylogenomic framework of Yang et al. [18], but in providing a densely sampled complete-plastome resource and a detailed organellar-genome perspective on structural evolution, mutational hotspots, candidate molecular markers, and lineage-specific plastid gene selection.

## Conclusion

This study expands previous Moraceae plastome studies by incorporating 49 newly sequenced plastomes and analyzing 140 complete plastomes, with 127 high-quality plastomes used for comparative genomic analyses. Moraceae plastomes are broadly conserved in genome structure, but lineage-specific IR boundary variation represents an important source of structural diversity. By classifying IR boundaries into four major types and 11 subtypes and reconstructing ancestral IR boundary states, our study refines current understanding of plastome structural evolution in the family. The highly variable non-coding regions and protein-coding genes identified here provide candidate molecular markers for future systematic and species-identification studies, while selection-pressure analyses suggest that most plastid genes are under purifying selection, with only a few genes showing lineage-specific signals of positive selection. The plastome-based topology largely corroborates current tribal classifications and supports several generic-level results, including the placement of *Maclura* within Chlorophoreae and the non-monophyly of *Streblus*. However, because this study is based solely on complete plastome sequences, the inferred topology should be interpreted as a plastome-based phylogeny rather than a comprehensive species-tree reconstruction. Thus, our results complement recent nuclear and plastid phylogenomic frameworks by providing a densely sampled complete-plastome resource and detailed insights into plastome structural evolution in Moraceae.

## Supplementary Information

Supplementary Material 1.

## Data Availability

The newly sequenced chloroplast genome sequences have been deposited in the CNGB Sequence Archive (CNSA) of the China National GeneBank DataBase (CNGBdb) under project number CNP0008966, with accession numbers N_002032133–N_002032181.
