# Supplementary material for: Comparative plastome analysis and plastomic phylogeny of Moraceae with expanded species-level sampling
Source: BMC Plant Biol. 2026 Jul 15;26:1736. doi: 10.1186/s12870-026-09508-4 (PMC13651133; doi:10.1186/s12870-026-09508-4)
Supplement: Supplementary file 1 [file 12870_2026_9508_MOESM1_ESM.zip › Supplementary_Figures.pdf]

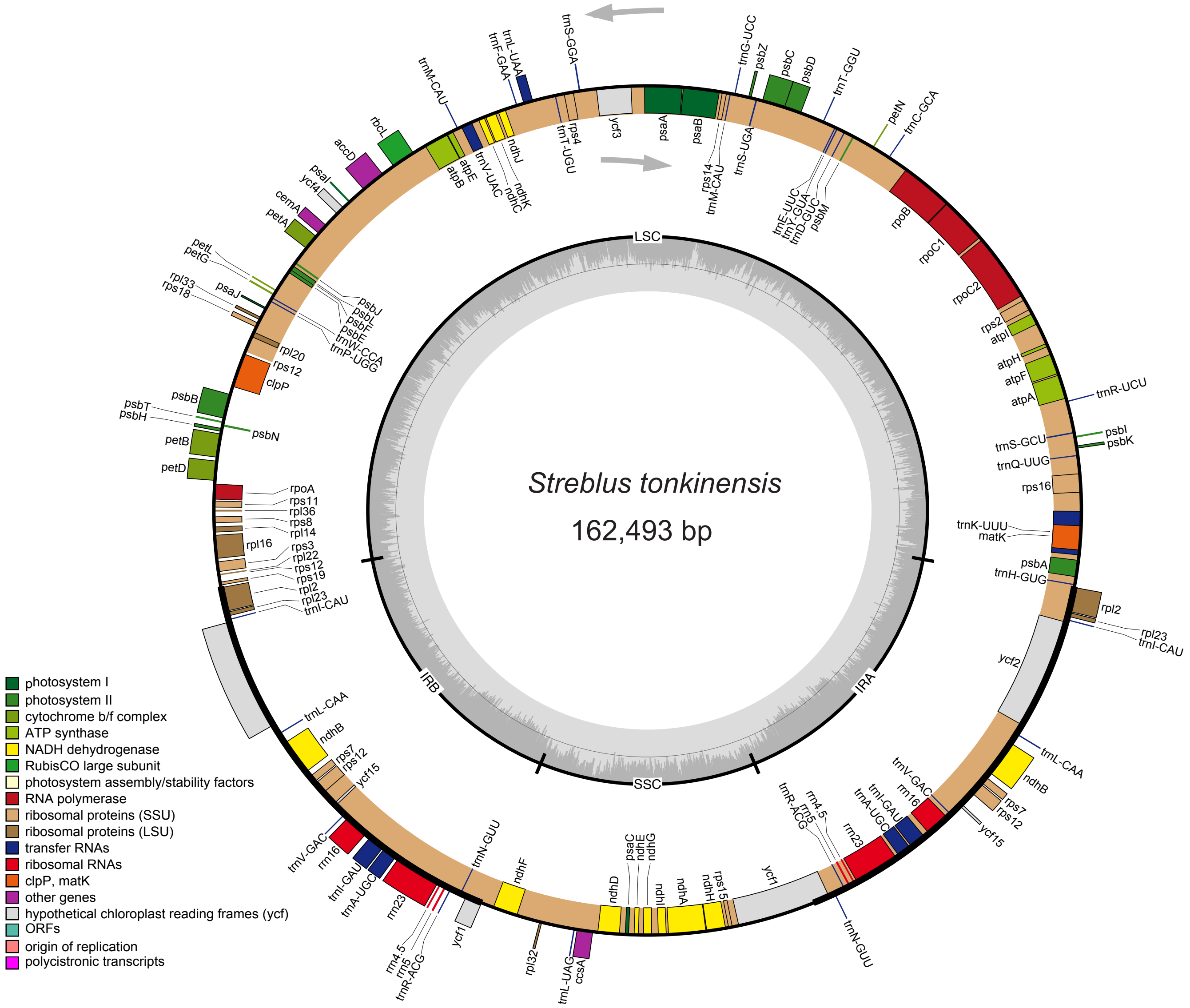

Figure S1 The circular plastome map of *Streblus tonkinensis*. Genes inside the circle are transcribed clockwise, while genes outside the circle are transcribed counterclockwise. Genes belonging to different functional groups are marked with different colors. The darker and lighter gray areas in the inner circle corresponds to the GC content and AT content, respectively.

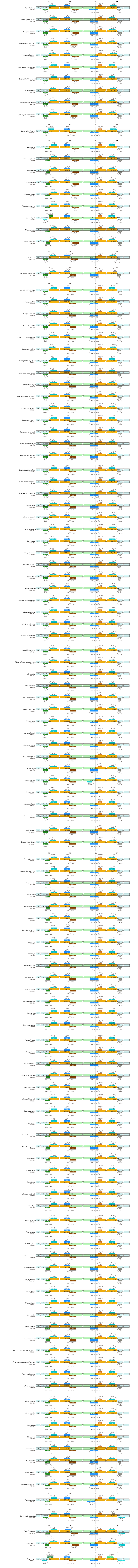

Figure S2 Comparative analysis of IR boundaries across all 127 Malvaceae samples.

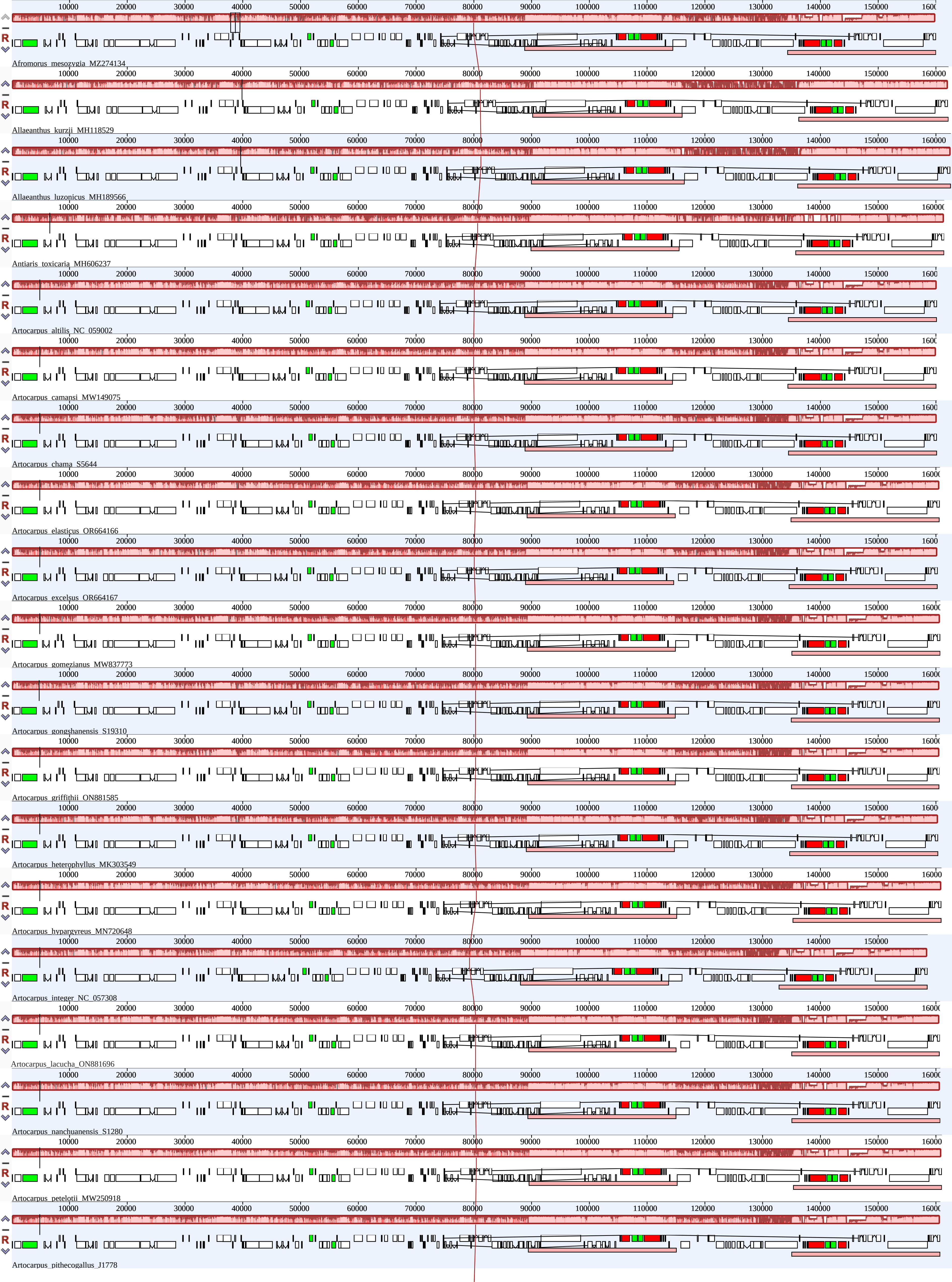
Afromorus_mesozygia_MZ274134
Allaeanthus_kurzii_MH118529
Allaeanthus_luzonicus_MH189566
Antiaris_toxicaria_MH606237
Artocarpus_altilis_NC_059002
Artocarpus_camansi_MW149075
Artocarpus_chama_S5644
Artocarpus_elasticus_OR664166
Artocarpus_excelsus_OR664167
Artocarpus_gomezianus_MW837773
Artocarpus_gongshanensis_S19310
Artocarpus_griffithii_ON881585
Artocarpus_heterophyllus_MK303549
Artocarpus_hypargyreus_MN720648
Artocarpus_integer_NC_057308
Artocarpus_lacucha_ON881696
Artocarpus_nanchuanensis_S1280
Artocarpus_petelotii_MW250918
Artocarpus_pithecogallus_J1778

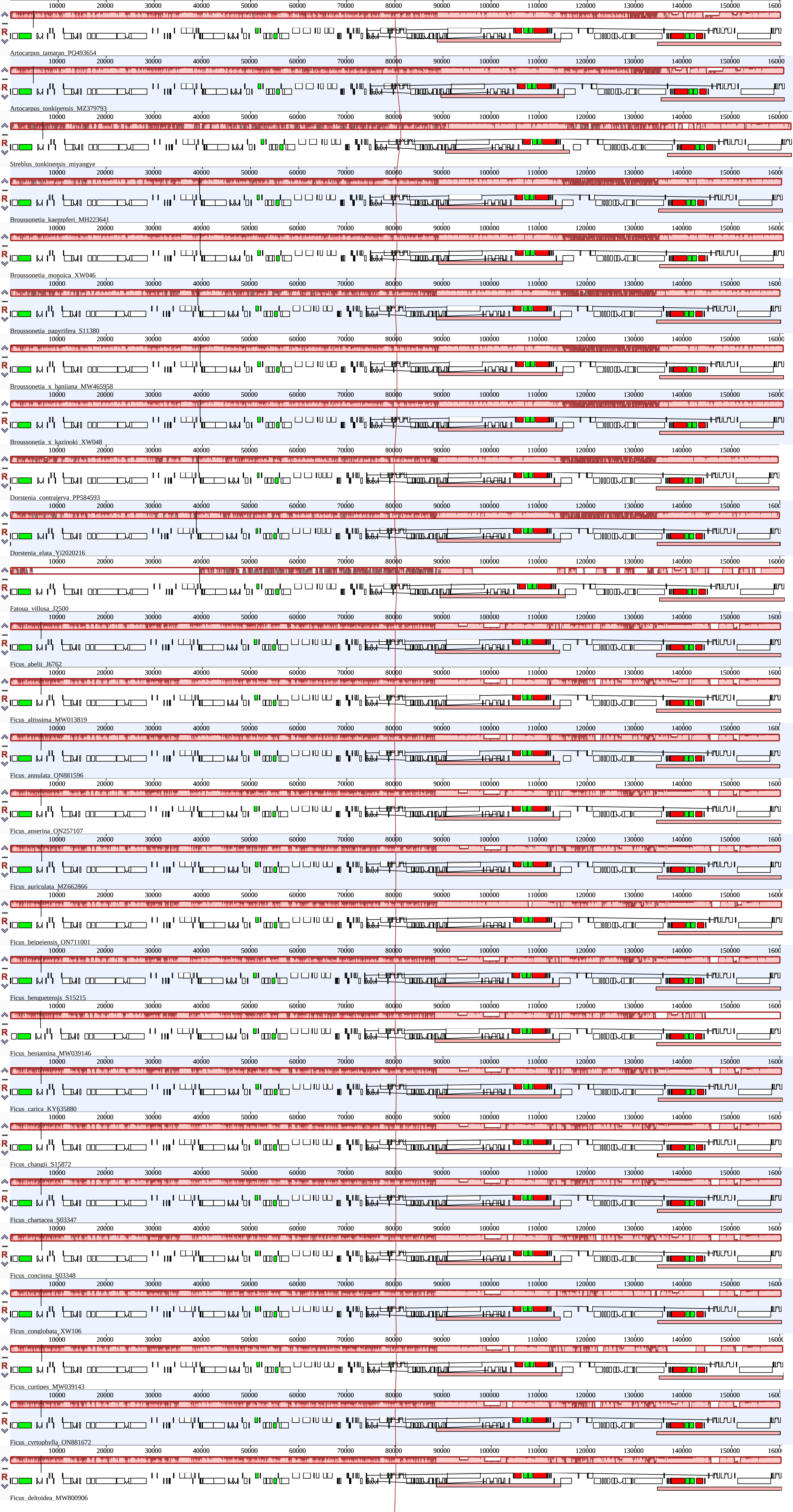
Artocarpus_tamaran_PQ493654
Artocarpus_tonkinensis_MZ379793
Streblus_tonkinensis_miyangye
Broussonetia_kaempferi_MH223641
Broussonetia_monoica_XW046
Broussonetia_papyrifera_S11380
Broussonetia_x_hanjiana_MW465958
Broussonetia_x_kazinoki_XW048
Dorstenia_contrajerva_PP584593
Dorstenia_elata_Yi2020216
Fatoua_villosa_J2500
Ficus_abelii_J6762
Ficus_altissima_MW013819
Ficus_annulata_ON881596
Ficus_anserina_ON257107
Ficus_auriculata_MZ662866
Ficus_beipeiensis_ON711001
Ficus_benguetensis_S15215
Ficus_benjamina_MW039146
Ficus_carica_KY635880
Ficus_changii_S15872
Ficus_chartacea_S03347
Ficus_concinna_S03348
Ficus_conglobata_XW106
Ficus_curtipes_MW039143
Ficus_cyrtophylla_ON881672
Ficus_deltoidea_MW800906

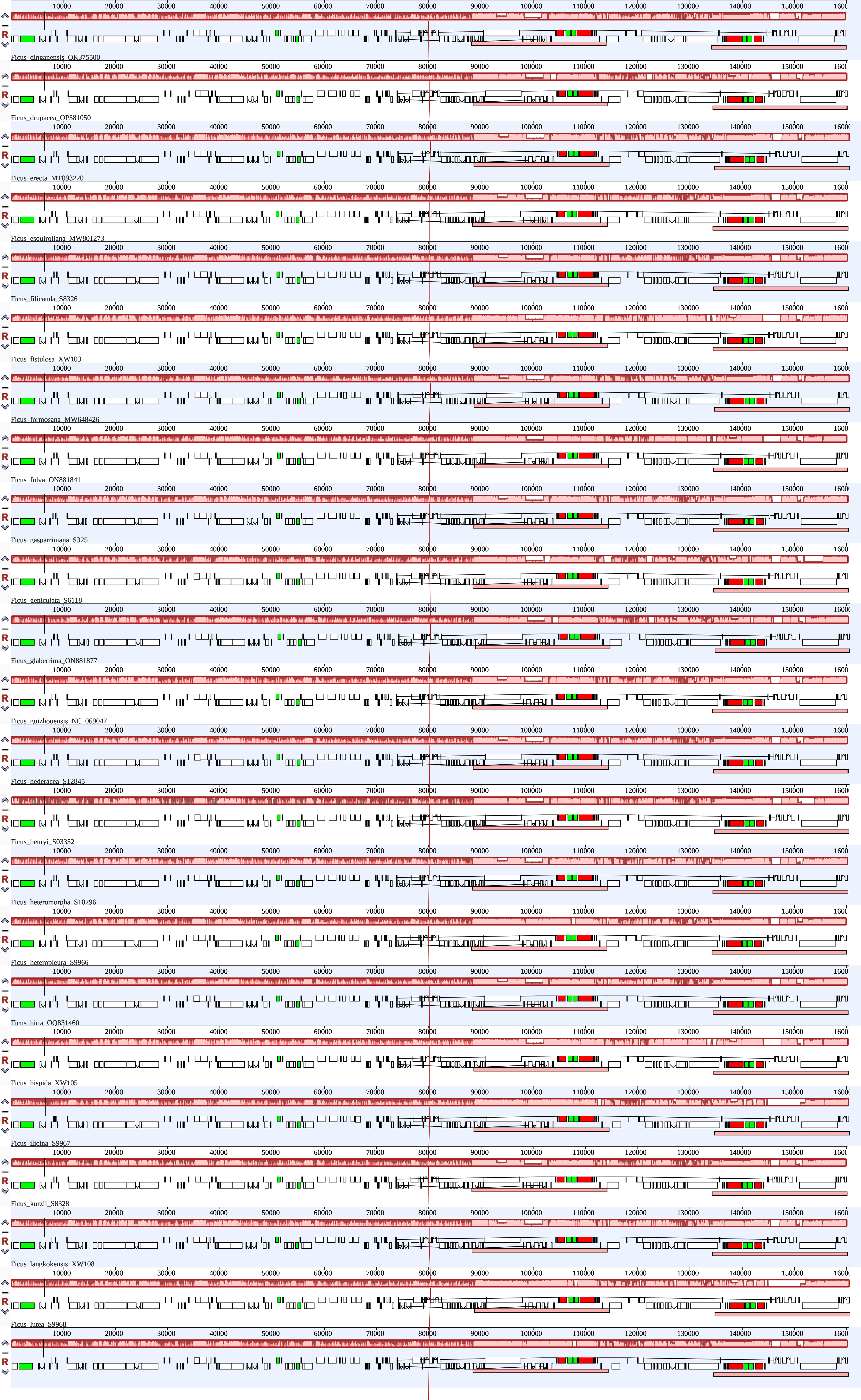
10000
20000
30000
40000
50000
60000
70000
80000
90000
100000
110000
120000
130000
140000
150000
Ficus_dinganensis_OK375500
Ficus_drupacea_OP581050
Ficus_erecta_MT093220
Ficus_esquiroliana_MW801273
Ficus_filicauda_S8326
Ficus_fistulosa_XW103
Ficus_formosana_MW648426
Ficus_fulva_ON881841
Ficus_gasparriniana_S325
Ficus_geniculata_S6118
Ficus_glaberrima_ON881877
Ficus_guizhouensis_NC_069047
Ficus_hederacea_S12845
Ficus_henryi_S03352
Ficus_heteromorpha_S10296
Ficus_heteropleura_S9966
Ficus_hirta_OQ831460
Ficus_hispida_XW105
Ficus_ilicina_S9967
Ficus_kurzii_S8328
Ficus_langkokensis_XW108
Ficus_lutea_S9968

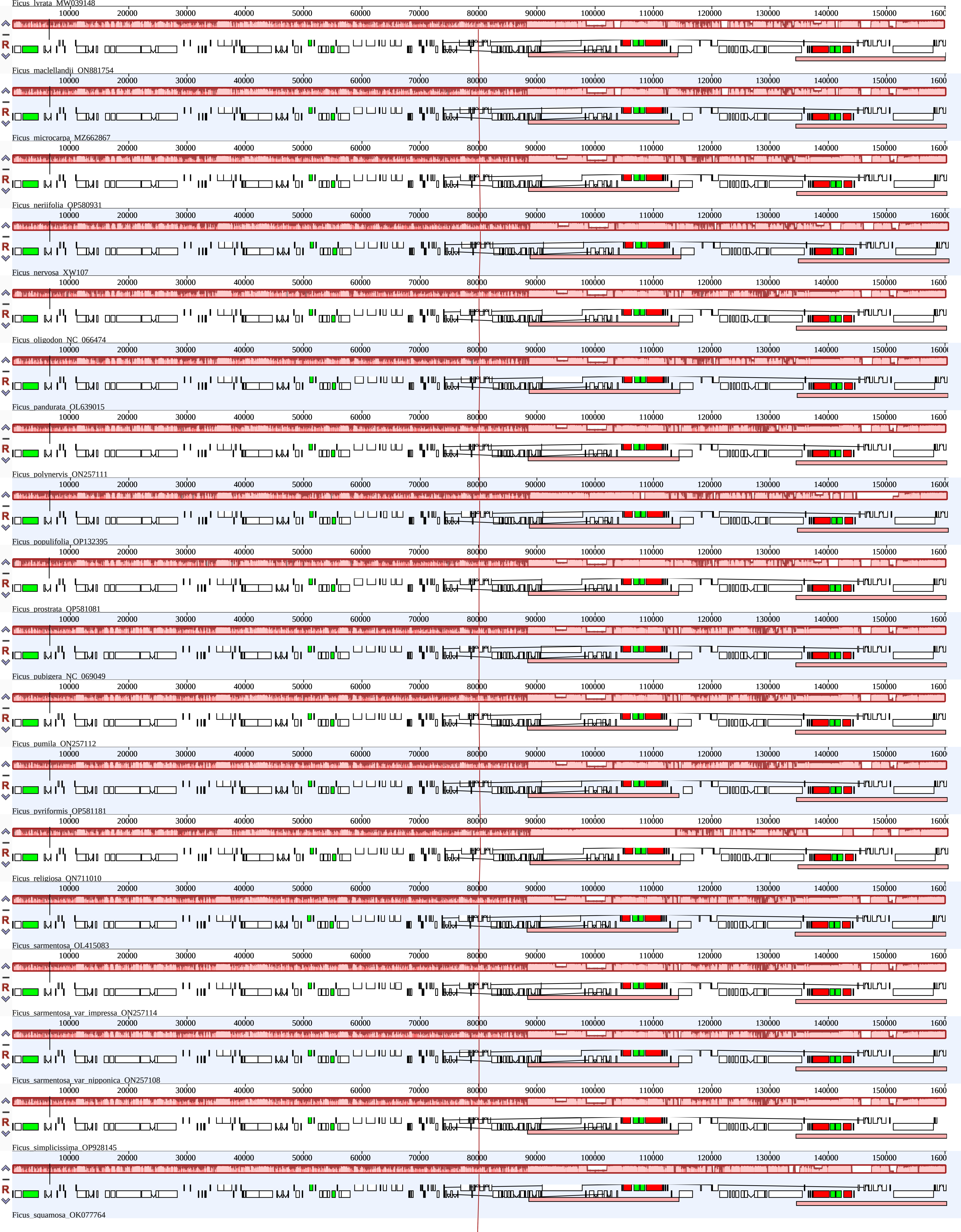
Ficus_lyrata_MW039148
Ficus_maclellandii_ON881754
Ficus_microcarpa_MZ662867
Ficus_neriifolia_OP580931
Ficus_nervosa_XW107
Ficus_oligodon_NC_066474
Ficus_pandurata_OL639015
Ficus_polynervis_ON257111
Ficus_populifolia_OP132395
Ficus_prostrata_OP581081
Ficus_pubigera_NC_069049
Ficus_pumila_ON257112
Ficus_pyriformis_OP581181
Ficus_religiosa_ON711010
Ficus_sarmentosa_OL415083
Ficus_sarmentosa_var_impressa_ON257114
Ficus_sarmentosa_var_nipponica_ON257108
Ficus_simplicissima_OP928145
Ficus_squamosa_OK077764

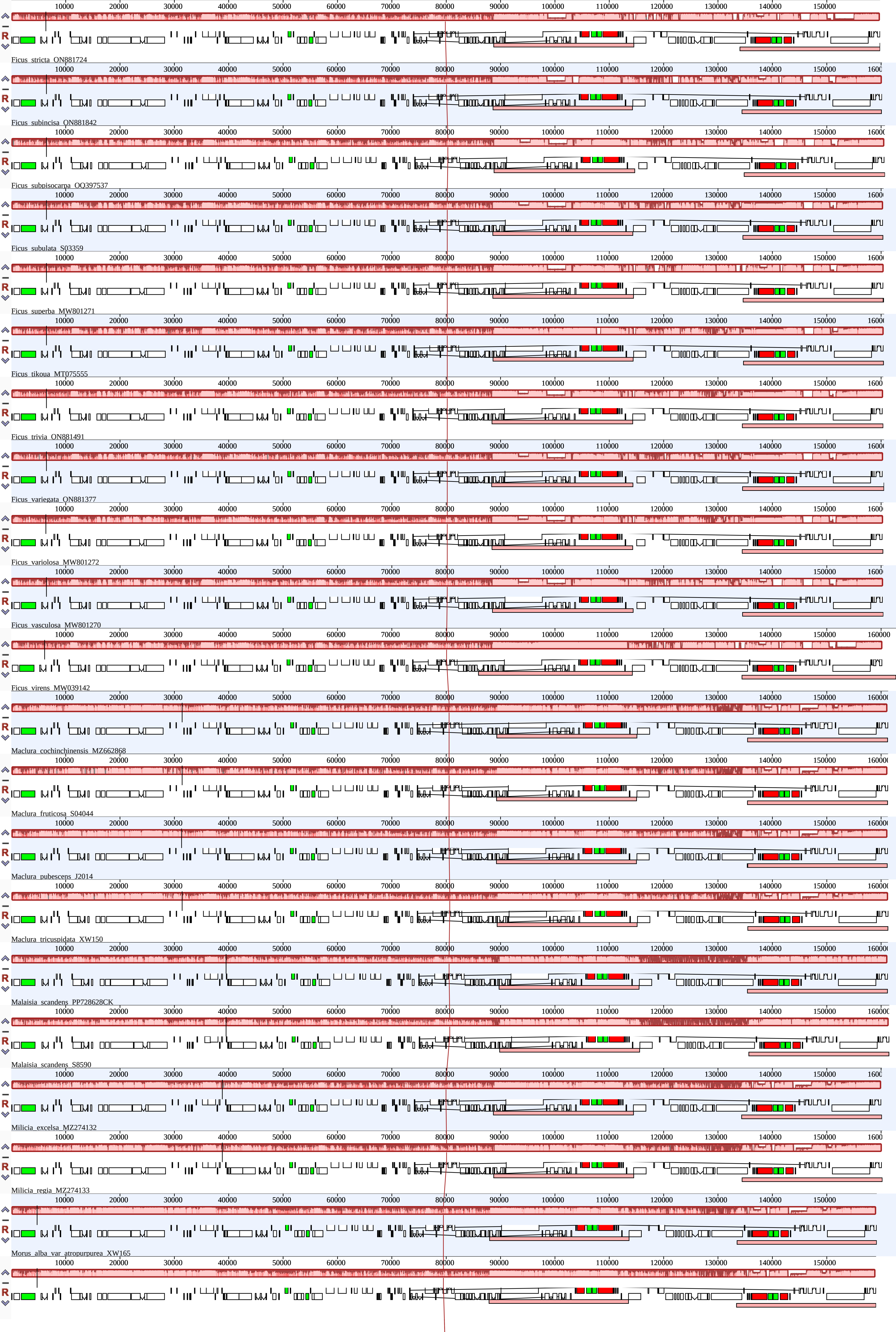
Ficus_stricta_ON881724
Ficus_subincisa_ON881842
Ficus_subpisocarpa_OQ397537
Ficus_subulata_S03359
Ficus_superba_MW801271
Ficus_tikoua_MT075555
Ficus_trivia_ON881491
Ficus_variegata_ON881377
Ficus_variolosa_MW801272
Ficus_vasculosa_MW801270
Ficus_virens_MW039142
Maclura_cochinchinensis_MZ662868
Maclura_fruticosa_S04044
Maclura_pubescens_J2014
Maclura_tricuspidata_XW150
Malaisia_scandens_PP728628CK
Malaisia_scandens_S8590
Milicia_excelsa_MZ274132
Milicia_regia_MZ274133
Morus_alba_var_atropurpurea_XW165

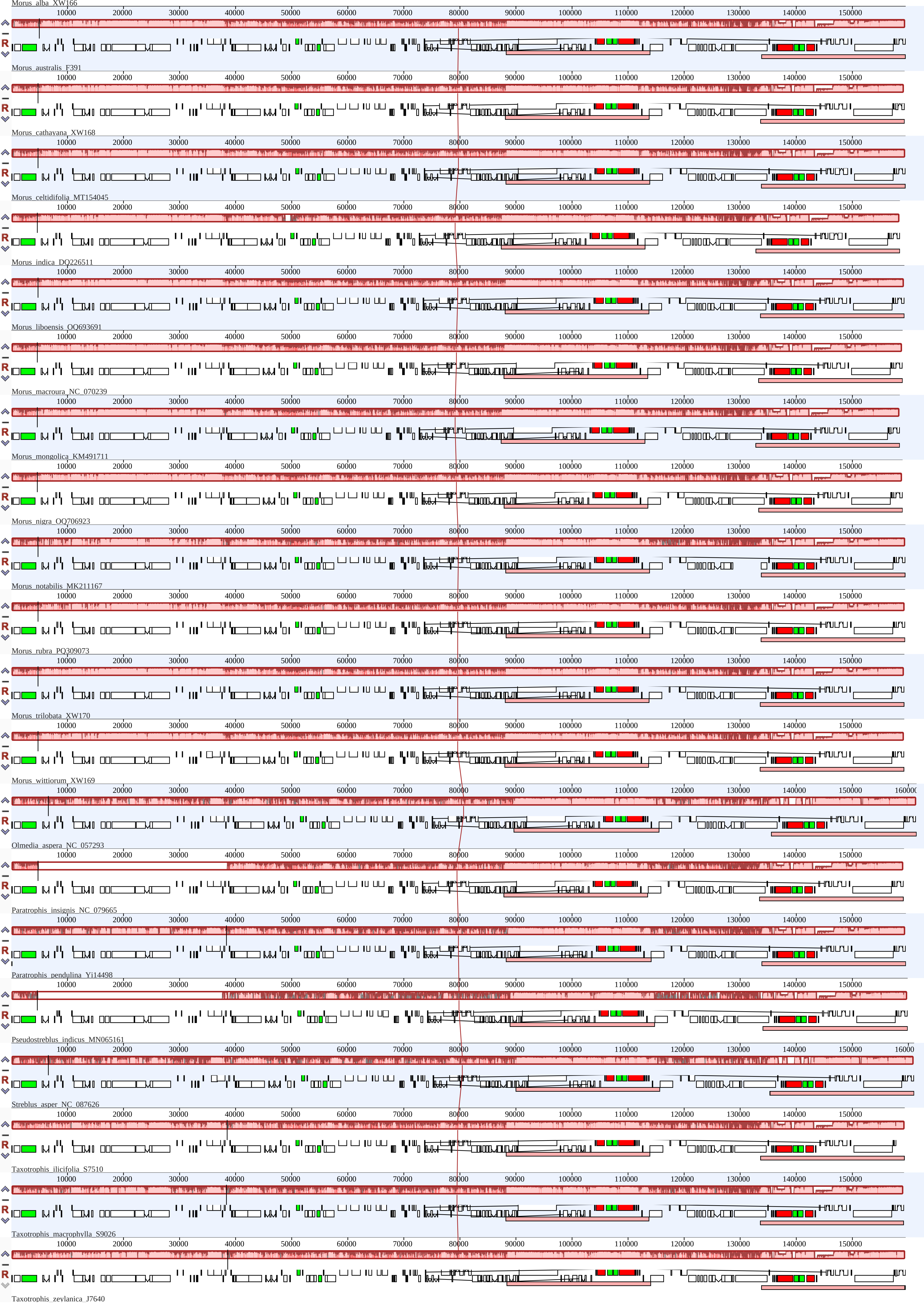

Figure S3 Collinearity analysis of 127 Moraceae plastomes.

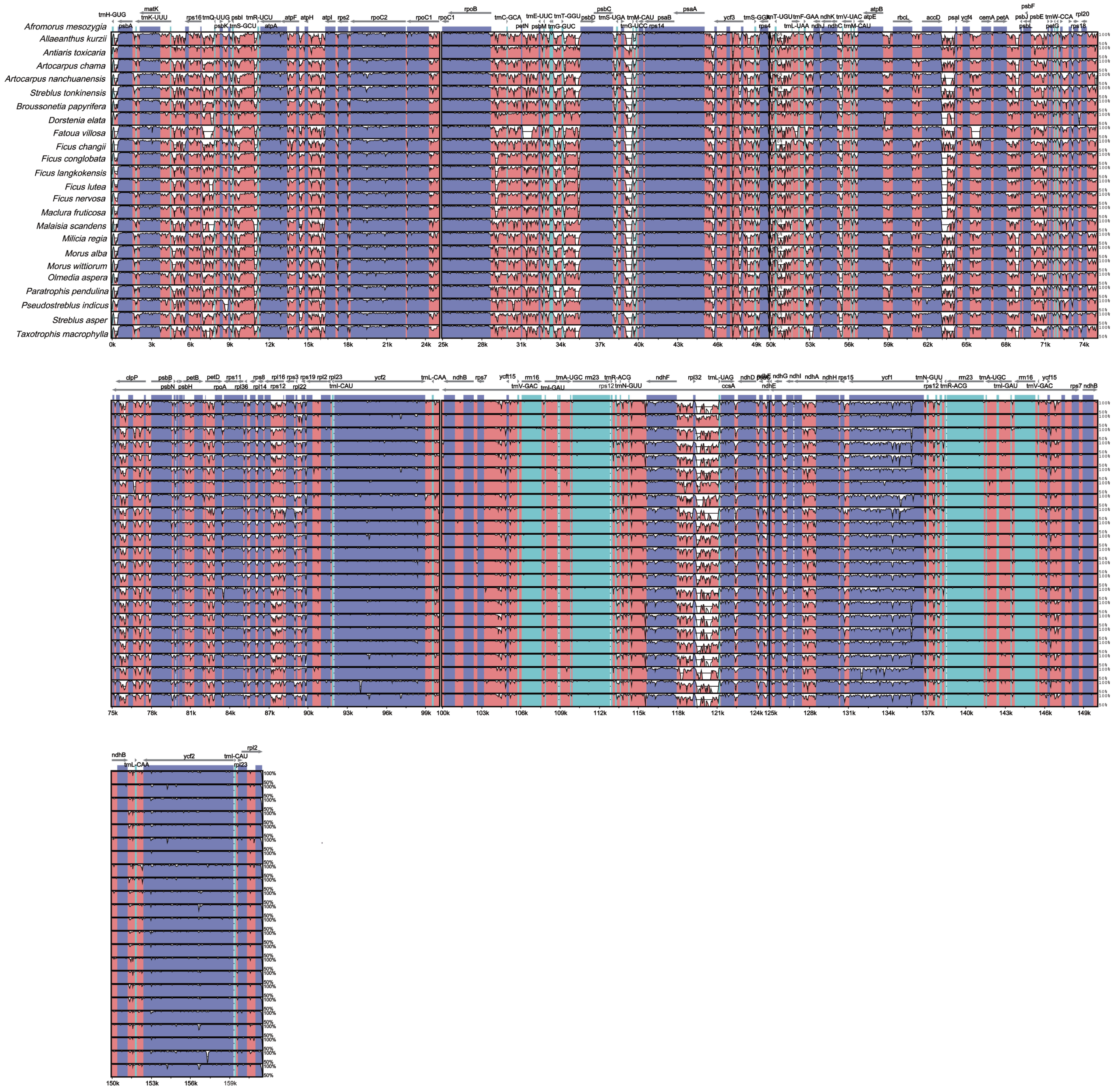

Figure S4 Sequence alignment visualization of 25 Moraceae plastomes using mVISTA.

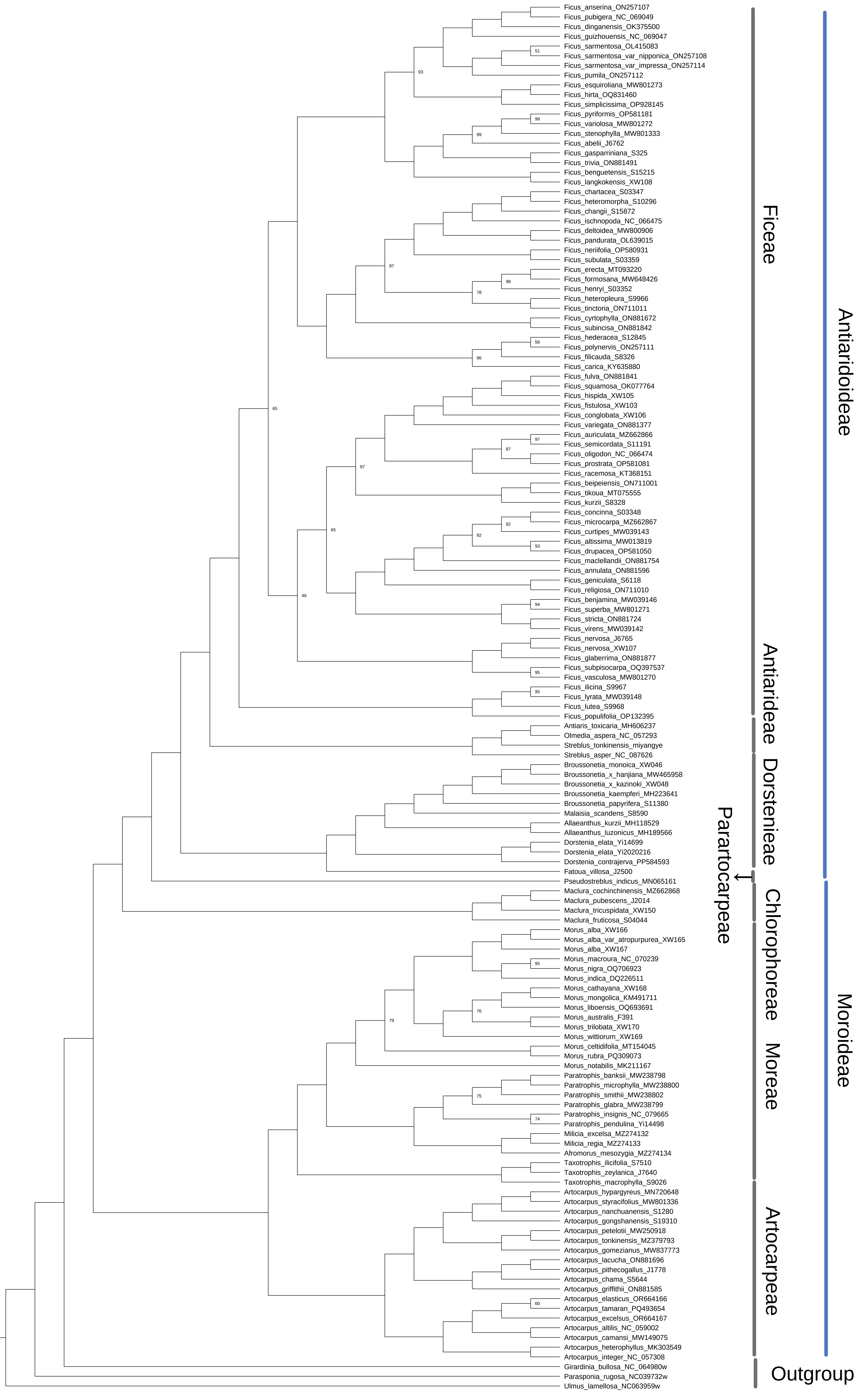

Figure S5A Phylogenetic tree inferred from concatenated CDS sequences. Bootstrap support values are shown only for nodes with <100% support; unlabeled nodes indicate 100% bootstrap support.

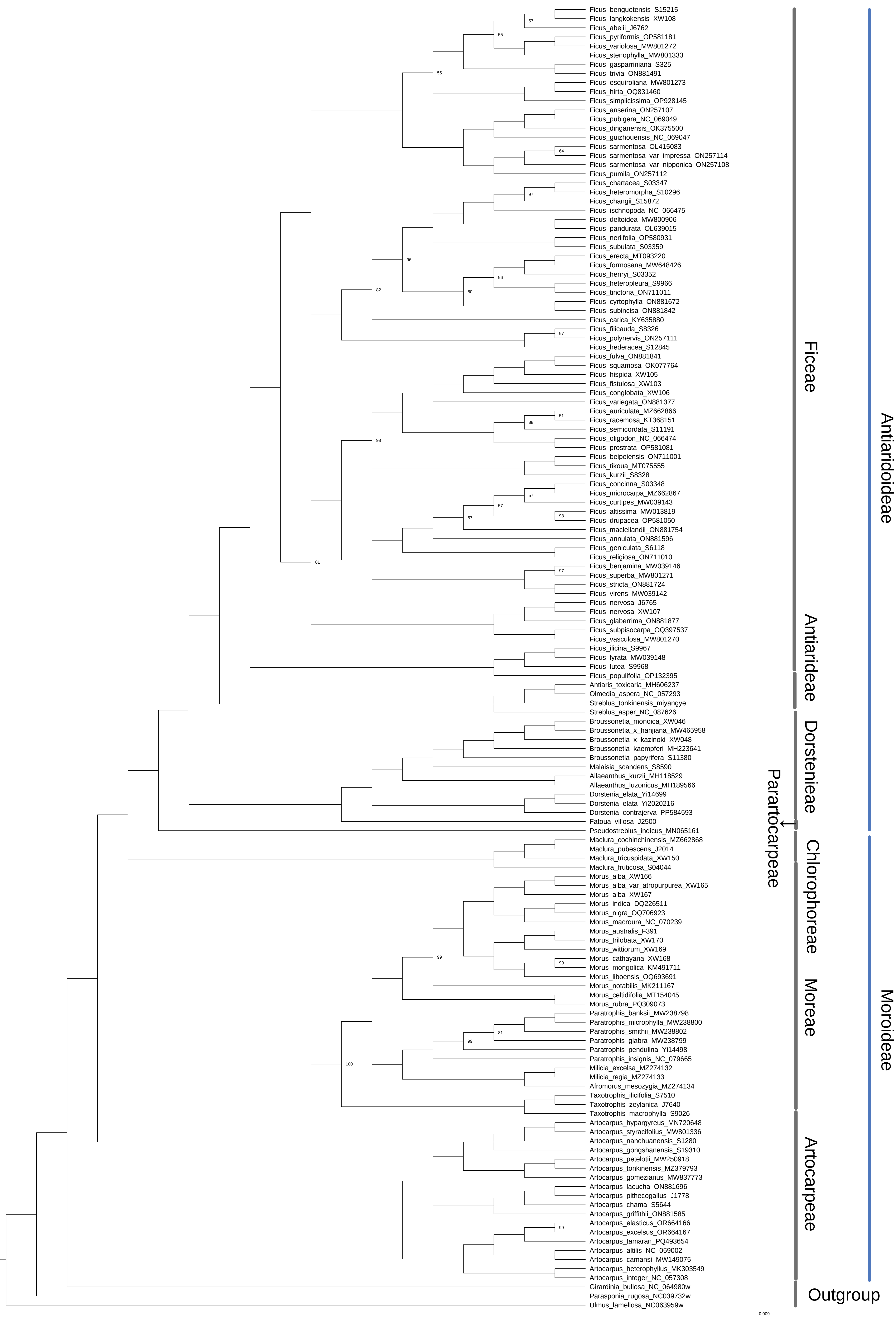

Figure S5B Phylogenetic tree inferred from concatenated IGS sequences. Bootstrap support values are shown only for nodes with <100% support; unlabeled nodes indicate 100% bootstrap support.
